# Cancer cell-intrinsic rewiring of the colorectal cancer secretome

**DOI:** 10.1016/j.isci.2026.117525

**Published:** 2026-09-22

**Authors:** Maartje Witsen, Theodoros Foskolos, Jelmer J. Dijkstra, Miguel Parra-Martinez, Joyce J.I. Catsman, Paula T. van Royen, Bob J. Ignacio, Onno Bleijerveld, Marije C. Kuiken, Alex van Vliet, Kim Monkhorst, Willemijn S.M. E. Theelen, Daniel Peeper, Joris van de Haar, Kimberly Bonger, Michiel Vermeulen, Emile E. Voest, Krijn K. Dijkstra

**Affiliations:** 1 Division of Molecular Oncology & Immunology, The Netherlands Cancer Institute, Amsterdam, the Netherlands; 2 Oncode Institute, Utrecht, the Netherlands; 3 Division of Gene Regulation, The Netherlands Cancer Institute, Amsterdam, the Netherlands; 4 Division of Synthetic Organic Chemistry, Radboud University, Nijmegen, the Netherlands; 5 Proteomics Facility, The Netherlands Cancer Institute, Amsterdam, the Netherlands; 6 Department of Pathology, The Netherlands Cancer Institute, Amsterdam, the Netherlands; 7 Department of Thoracic Oncology, The Netherlands Cancer Institute, Amsterdam, the Netherlands; 8 Leiden Institute for Chemistry, Chemical Biology & Immunology, Leiden University, Leiden, the Netherlands; 9 Department of Molecular Biology, Faculty of Science, Radboud Institute for Molecular Life Sciences, Radboud University, Nijmegen, the Netherlands

**Keywords:** CRC, secretome, organoids, immune evasion, NOTUM

## Abstract

Secreted factors are essential mediators of cellular communication that are exploited by tumors to control their environment and evade immune attack. However, how cancer cells rewire their secretome is unclear due to challenges in reliably profiling cancer cell-specific secreted factors. Here, we apply a click chemistry-based approach to selectively profile the secretome of patient-derived colorectal cancer (CRC) and healthy colon organoids. We observe cancer cell-intrinsic rewiring of the secretome during malignancy, with clear separation between microsatellite stable (MSS) and instable cancers. We then leverage this secretomics dataset to identify secreted factors that mark immune-excluded cancers, revealing NOTUM as a marker of decreased T cell infiltration across four independent datasets. Overall, our study provides an in-depth characterization of the cancer cell-intrinsic secretome of MSS-CRC and nominates NOTUM as a secreted factor marking immune-excluded colorectal cancers. This provides a scalable framework to link cancer cell-derived secreted factors to relevant tumor phenotypes.

## Highlights

• Click chemistry-based method maps cancer cell-intrinsic secretomes in CRC organoids
• The MSS-CRC cell-intrinsic secretome is rewired in transition to malignancy
• MSS and MSI colorectal cancers show distinct secretory profiles
• NOTUM is a secreted factor marking immune-excluded MSS-CRC tumors

## Graphical abstract

**Figure:**
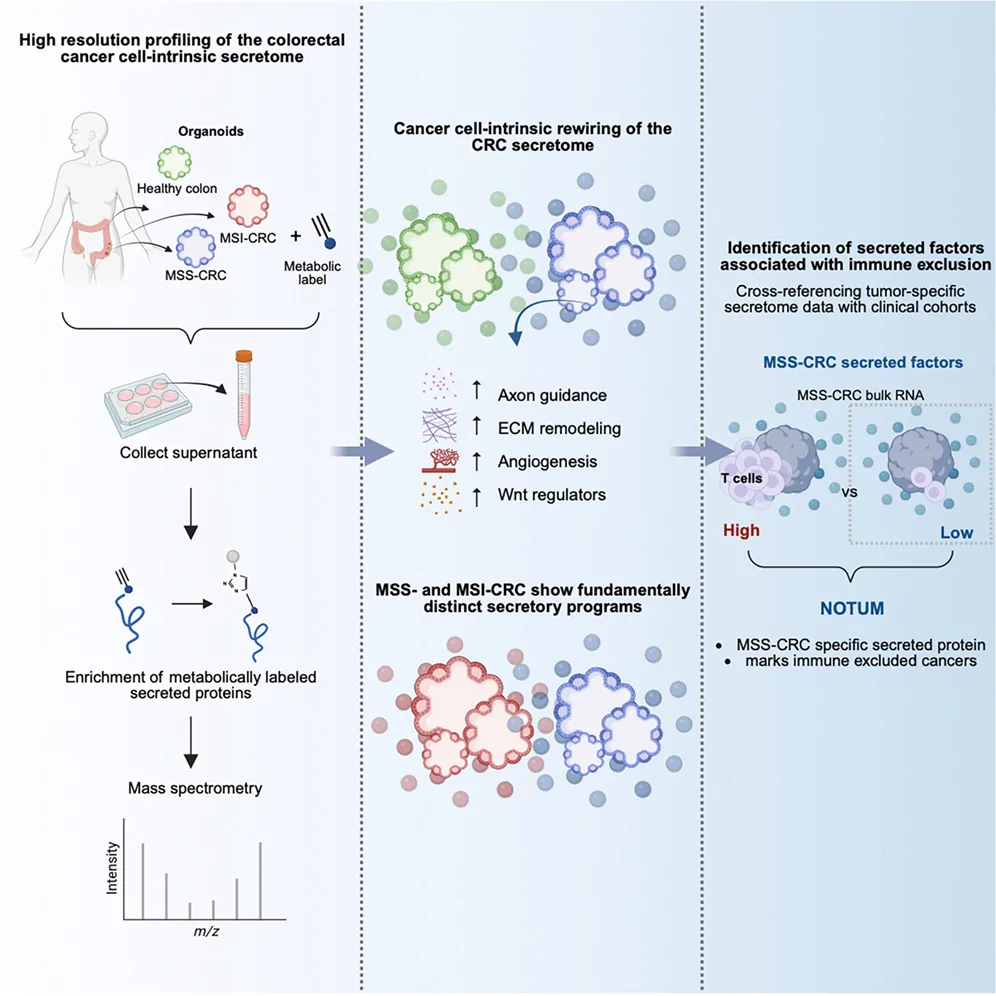

## Teaser

Oncology; Systems biology; Cancer systems biology; Cancer

## Introduction

Cancer cells control their microenvironment to promote their growth and dissemination within the body, which is primarily mediated via either cell-cell contact or secreted factors. Secreted factors are highly potent signaling molecules as they can control cells beyond their immediate vicinity, acting both at short and long range to shape tissues, attract immune cells and exert systemic effects.

This is particularly relevant in the context of coordinating immune crosstalk, which is exploited by tumors to avoid immune destruction. The majority (85%) of colorectal cancer (CRC) patients harbor microsatellite stable (MSS) tumors, which exhibit resistance to immune checkpoint inhibitors (ICIs) through various mechanisms.^1^^,^^2^ In addition to the low abundance of neoantigens, these include the establishment of an immune-suppressive TME and the scarcity of tumor infiltrating lymphocytes (TILs).^3^^,^^4^^,^^5^^,^^6^^,^^7^^,^^8^ As a result, patients with MSS-CRC often have limited therapeutic options and poor prognosis, especially in advanced stages. This highlights the need for alternative treatment strategies that enhance the immunogenicity of these tumors. However, despite the crucial role of secreted molecules in coordinating a favorable tumor microenvironment (TME), our understanding of how cancer cells alter their secretome compared to normal epithelial cells to promote their growth and avoid immune attack is largely unclear.

Several challenges have limited our ability to define the cancer cell-intrinsic secretome at high resolution. First, investigating cancer cell-specific secreted factors requires direct comparison with the secretome of healthy cells, which is difficult to achieve using conventional cancer cell lines. Furthermore, profiling of bulk tissues^9^^,^^10^^,^^11^^,^^12^^,^^13^ makes it impossible to resolve whether proteins are secreted by cancer cells or non-malignant cells. Although single-cell RNA sequencing can identify cellular sources of secreted factors, mRNA abundance poorly reflects protein levels and cannot reliably quantify secreted protein output.^13^^,^^14^^,^^15^ Last, conventional secretomics approaches have limited sensitivity for the detection of secreted proteins since secreted proteins are often lowly abundant and masked by highly abundant serum- or media-derived proteins.

Here, we overcome these limitations by applying recent innovations in secretomics profiling to patient-derived normal and tumor organoids. Organoids can be established from both colorectal cancer and normal epithelium of the same patient, allowing comprehensive profiling of the cancer cell-intrinsic secretome in models directly established from patient tissues. To enhance the resolution of secretome profiling, several metabolic protein labeling approaches have been developed based on bioorthogonal methionine or puromycin analogs that enable selective enrichment of newly synthesized proteins. However, these protein labeling methods often require methionine-free culture conditions, auxotrophic cell systems, and/or can induce cellular toxicity, which is challenging for patient-derived organoid models that grow in specialized media.^16^^,^^17^^,^^18^ Here, we apply “threonine-derived non-canonical amino acid tagging” (THRONCAT),^19^ a recently developed non-toxic metabolic labeling method that enables detection of secreted proteins in complete culture media with high sensitivity. In short, incorporation of the threonine analog β-ethynylserine (βES) allows fast metabolic labeling of newly synthesized proteins, which can be selectively enriched from culture media via click chemistry and quantified by mass spectrometry (MS). By applying THRONCAT on tumor and healthy organoids directly established from patients, we can now directly measure the cancer cell-intrinsic secretome with high resolution.

Focusing on the poorly immunogenic MSS subtype of CRC, we aimed to define the cancer cell-intrinsic secretome of MSS-CRC organoids and identify secreted factors associated with immune-excluded cancers. Using THRONCAT on healthy colon (HC) and MSS-CRC organoids, we show a major rewiring of the CRC secretome compared to normal colon organoids. Notably, the secretome of MSS-CRC is markedly distinct from that of the more immune-sensitive microsatellite stable instable (MSI) subtype. Next, we cross-referenced our secretomics data with RNA sequencing of tumors with high and low T cell activity and identified NOTUM as a secreted factor associating with poor T cell infiltration in multiple independent datasets. Collectively, our approach provides an in-depth characterization of the altered CRC secretome and the identification of secreted factors associated with immune-excluded tumors.

## Results

### Cancer cell-intrinsic rewiring of the CRC secretome

The extent to which malignant transformation alters the cellular secretome is unclear. We used THRONCAT^19^ to profile the secretome of healthy and tumor organoids using high resolution quantitative MS (Table 1; Figure 1A). To confirm the optimal time window for labeling of newly synthesized proteins, we performed a pilot experiment, which demonstrated an increased number of detected and differentially expressed proteins after 24 h compared to 5 h of labeling (Figures S1A and S1B). Treatment with 0.5 mM of the threonine analog βES to label newly synthesized proteins did not induce detectable toxicity at 24 h or even 48 h, indicating that it is well tolerated in organoids (Figures S1C and S1D).

**Table 1:** Characteristics of CRC and HC organoids

Organoid line	Tumor or healthy tissue	MSI status	Primary tumor or metastasis	Biopsy or resection	Tumor location	Sex	Age (years)	Analyses	ELISA FGF19	ELISA NOTUM
ITO-045T	tumor	MSI	primary	resection	colon	female	76	Figure 2	X	–
ITO-058T	tumor	MSI	primary	resection	colon	female	71	Figure 2	X	–
ITO-058M	tumor	MSI	metastasis	resection	locoregional recurrence	female	71	Figure 2	X	–
ITO-079T	tumor	MSI	primary	resection	colon	female	85	Figure 2	X	–
ITO-111Tpre^a^	tumor	MSI	metastasis	biopsy	peritoneum	female	83	Figure 2	X	–
ITO-111Ton^b^	tumor	MSI	primary	biopsy	colon	female	83	Figure 2	X	–
ITO-008T	tumor	MSS	metastasis	resection	liver	male	46	Figures 1 and 2	X	X
ITO-014Tb	tumor	MSS	metastasis	resection	liver	female	84	Figure 2	X	–
ITO-055T	tumor	MSS	primary	resection	rectum	male	65	–	–	X
ITO-060T	tumor	MSS	primary	resection	colon	female	55	Figures 1 and 2	X	X
ITO-063T	tumor	MSS	primary	resection	colon	female	78	Figures 1 and 2	X	–
ITO-108T	tumor	MSS	metastasis	biopsy	liver	male	64	Figure 2	X	–
ITO-113T	tumor	MSS	primary	resection	colon	male	59	Figures 1 and 2	X	–
ITO-118T	tumor	MSS	primary	resection	rectum	female	79	–	–	X
ITO-180T	tumor	MSS	primary	resection	rectosigmoid	female	70	–	–	X
ITO-179T	tumor	MSS	primary	resection	rectum	male	69	Figure 2	X	–
ITO-044H	healthy	–	–	resection	colon	male	72	–	–	X
ITO-063H	healthy	paired MSS	–	resection	colon	female	78	Figure 1	–	–
ITO-113H	healthy	paired MSS	–	resection	colon	male	59	Figure 1	–	–
ITO-117H	healthy	paired MSI	–	resection	colon	female	74	Figure 1	–	X

Overview of organoid lines used in this study.

a Biopsy pre treatment with immunotherapy.

b Biopsy on treatment with immunotherapy.

**Figure 1:**
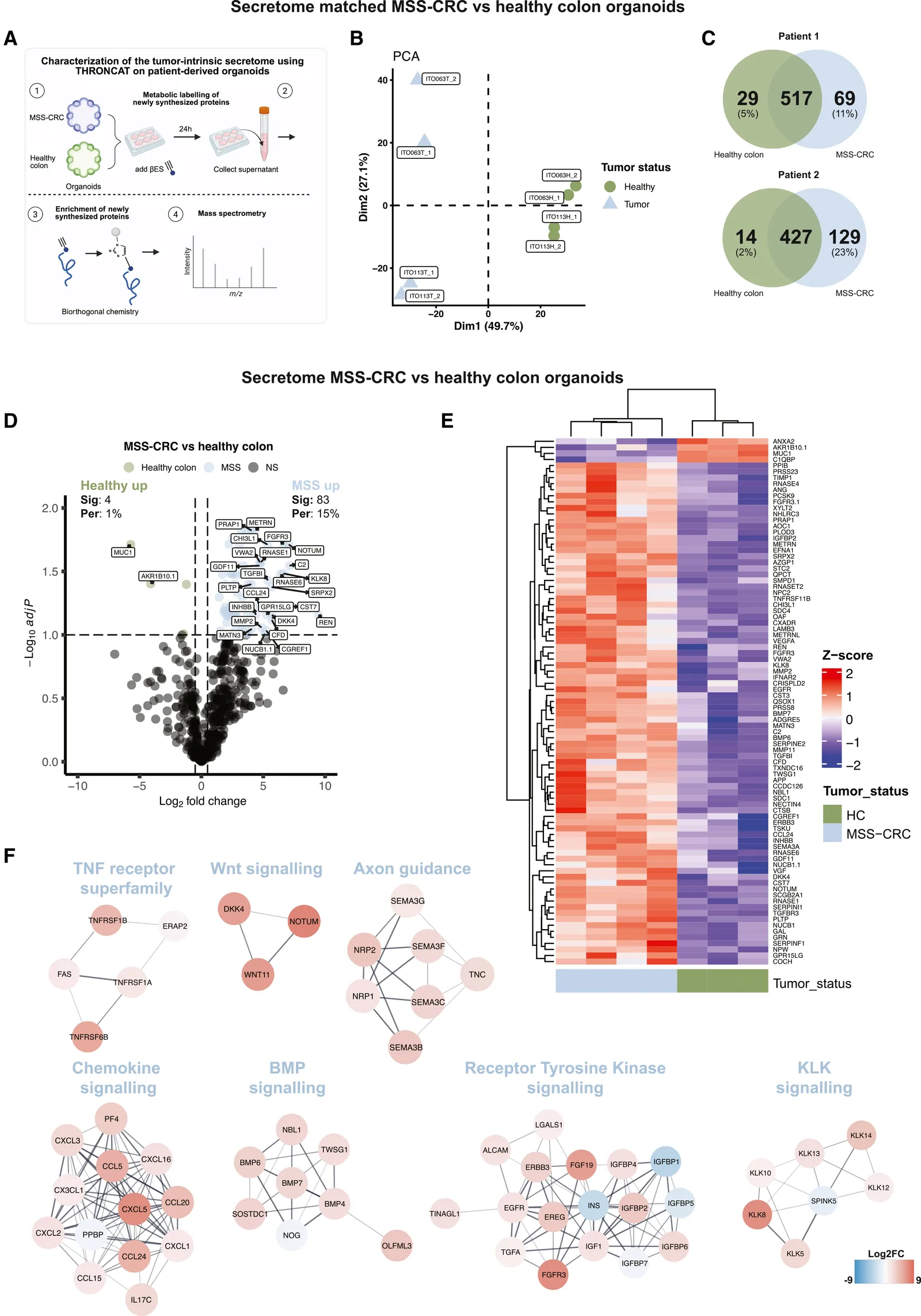
Cancer cell-intrinsic rewiring of the CRC secretome (A) Workflow to detect newly synthesized proteins produced by organoids using THRONCAT (threonine-derived non-canonical amino acid tagging method). To label newly synthesized proteins, the bioorthogonal threonine analog β-ethynylserine (βES) was added to the culture media for 24 h. Thereafter, newly synthesized proteins were isolated from the supernatant using click-chemistry and measured by LC-MS/MS. Figure created using Biorender.com. (B) PCA showing the first two principal components based on the secretomics values from paired healthy colon (HC) and CRC organoids from 2 patients (two independent replicates per organoid line). (C) Venn diagram showing the number of overlapping and unique differentially expressed secreted proteins (|log2FC| > 1, adj *p* <0.05) in MSS-CRC vs. HC organoids (*n* = 2) from patient 1 and 2. Significance was based on differential expression analysis using Student’s *t* test and adjusted for multiple testing using the Benjamini-Hochberg method. (D) Volcano plot showing differentially expressed secreted proteins in MSS-CRC (*n* = 4) vs. HC (*n* = 3) organoids (adjusted *p* value < 0.1). “Sig” indicates number of significant proteins, “per” indicates percentage of significant proteins of all detected proteins. Significance was based on differential expression analysis using a mixed linear model correcting for replicates and adjusted for multiple testing using the Benjamini-Hochberg method. (E) Heatmap showing *Z* score of significant proteins from (D) in MSS-CRC (*n* = 4) vs. HC (*n* = 3) organoids (average from two independent replicates per organoid line). (F) Selection of clusters from STRING protein interaction network on the secretomics dataset MSS-CRC (*n* = 4) vs. HC (*n* = 3) organoids, only nodes with |log2FC| >0.58 are shown. Color indicates log2FC. Full set of clusters shown in Figure S3F.

To directly compare the secretome of healthy and tumor-derived samples, unaffected by differences in germline or life history between patients, we analyzed the secretome of two pairs of HC and tumor-derived organoids from the same patient using 24 h of labeling. We focused our analysis on proteins annotated as secreted by the human protein atlas to filter out noise due to release of intracellular proteins into the culture media during cellular turnover. After filtering, 615 and 517 proteins were identified for patient 1 and patient 2, respectively.^20^ Principal component analysis (PCA) clearly separated HC and CRC organoids from both patients based on their secretome (Figure 1B). Rewiring of the MSS-CRC secretome compared to HC organoids was observed for multiple secreted proteins, with as much as 16%–25% of their secretome significantly altered (Figures 1C, S2A, and S2B).

To comprehensively study secretome rewiring in MSS-CRC, we next compared the secretome of a larger cohort of CRC and healthy organoids (Table 1). After filtering for proteins annotated as secreted, 559 proteins were identified (Table S1). Again, PCA demonstrated rewiring of the MSS-CRC secretome compared to that of HC organoids (Figure S3A). Similar trends were observed when performing PCA on the whole dataset (not filtered for proteins annotated as secreted) (Figure S3B), demonstrating that separation of tumor and normal samples based on their secretome is robust and independent of the filtering strategy. Analysis of the loadings of principal component (PC) 1 (the PC separating MSS-CRC vs. HC), shows that multiple proteins contribute to the separation of MSS-CRC vs. HC in the PCA (Figures S3C and S3D). This suggests that the differences between MSS-CRC and HC organoid secretomes are driven by several features, rather than being driven by only a small number of strongly changing proteins. Differential expression analysis revealed 83 significantly upregulated proteins, whereas 4 proteins were significantly downregulated in the secretome of MSS-CRC organoids (FDR <0.1; Figures 1D and 1E; Table S1). Analysis with alternative settings, including a stricter protein inclusion and alternative imputation strategy, showed strong agreement with the original analysis, demonstrating that the key findings are robust across different analytical settings (Figure S3E).

We analyzed proteins significantly enriched in the MSS-CRC secretome and examined clusters of upregulated proteins using STRING network analysis (Figures 1D–1F and S3F). The MSS-CRC specific secretome was dominated by extracellular matrix (ECM) remodeling proteins (TIMP1, TGFBI, CTSB, LAMB3, PLOD3, VWA2, MMP2, and MMP11), proteins involved in angiogenesis (VEGFA, ANG, REN, EFNA1, and SRPX2) and neurotrophic proteins and axon guidance molecules (SEMA3A, SERPINF1, APP, SERPINI1, SERPINE2, GDF11, and METRN), the latter of which have—aside from their neurological functions—been associated with poor prognosis and liver metastasis.^21^^,^^22^^,^^23^ Furthermore, we observed clusters involved in regulating the colorectal stem cell niche, including bone morphogenetic proteins (BMP), and Wnt and receptor tyrosine kinase (RTK) signaling.^24^^,^^25^

Interestingly, several factors related to immune regulation were altered in MSS-CRC compared to HC organoids. This included chemokine signaling, increased expression of complement proteins and their regulators (C2, CFD, SRPX2; normally mainly produced by hepatocytes), as well as several TNF superfamily receptors, which were released through ectodomain shedding (Figure S3G) and can act as decoy receptors.^26^ Notably, we observed strong upregulation of negative regulators of Wnt signaling including DKK4, and most prominently NOTUM as one of our top hits. Dickkopf (DKK) proteins have been described to promote immune evasion by inhibiting natural killer (NK) cells and CD8^+^ T cells within tumors.^27^^,^^28^^,^^29^

Altogether, these data reveal extensive cancer cell-intrinsic rewiring of the CRC secretome compared to normal colon epithelium and establish a high-resolution patient-derived secretomics dataset that enables the systematic analysis of tumor-derived secreted proteins.

### MSS and MSI colorectal cancers show distinct secretory profiles

Colorectal cancers are classified into MSS or MSI subtypes, which differ profoundly in tumor biology, therapy responsiveness and immunogenicity. We therefore characterized the secretome of an expanded cohort of both MSS-CRC and MSI-CRC organoids (Table 1; Figure 2A) and identified 457 proteins after filtering for proteins annotated as secreted (Table S2). Interestingly, MSS- and MSI-CRC organoids formed clearly separated clusters upon unsupervised hierarchical clustering (Figure 2A) and PC analysis in both the filtered and unfiltered dataset (Figures 2B and S4A), particularly along the second PC (Figures S4B and S4C). A signature based on the negative-contributing genes along PCA dimension 2 also separated MSS and MSI cancers in bulk RNA sequencing datasets of primary (The Cancer Genome Atlas [TCGA]) and metastatic (Hartwig Medical Foundation [HMF]) CRC (Figures 2C and 2D). This supports the notion that the cancer cell-intrinsic secretome differs fundamentally between MSS and MSI colorectal cancers and validates the subtype-specific secretory programs identified in organoids.

**Figure 2:**
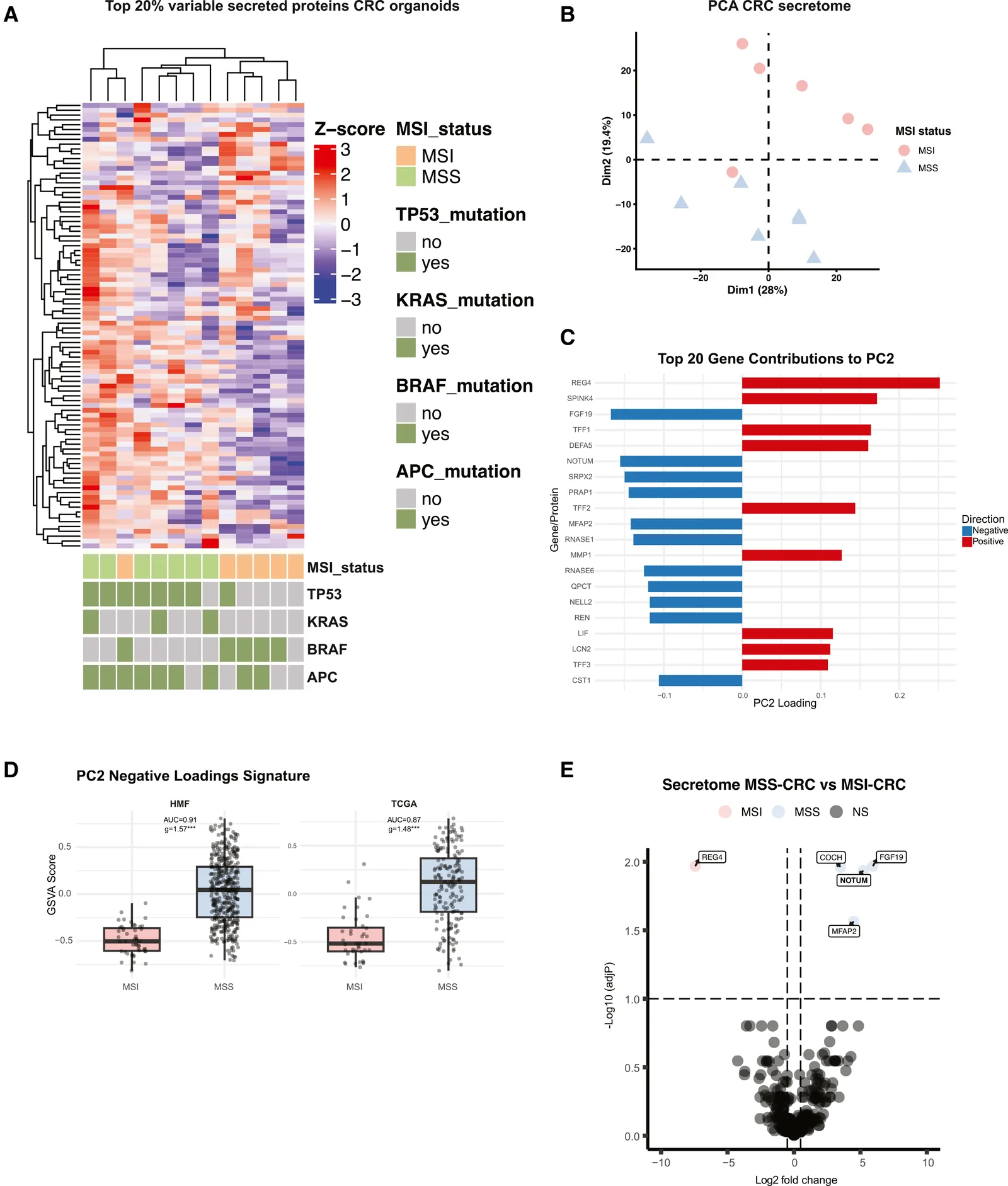
Microsatellite stable and instable colorectal cancers show distinct secretory profiles (A) Heatmap showing *Z* score of top 20% variable secreted proteins in MSS-CRC (*n* = 7) and MSI-CRC (*n* = 6) organoids (average from three independent replicates per organoid line). (B) PCA showing the first two principal components based on the secretomics values from MSS-CRC (*n* = 7) and MSI-CRC (*n* = 6) organoids (average from three independent replicates per organoid line). (C) Top 20 genes contributing to variance to PC2 in the PCA in (B). Negative contributors are marked in blue and positive contributors are marked in red. (D) Gene set variation analysis (GSVA) showing a per-sample enrichment score for top 10 negative contributors to PC2 (C) in MSS- and MSI-CRC samples in TCGA and HMF. AUC indicates area under the curve score and g indicates the effect size. Three (∗∗∗) indicate *p* ≤ 0.001. Boxes, whiskers, and dots indicate quartiles, 1.5 interquartile ranges, and individual tumors, respectively. (E) Volcano plot showing differentially expressed secreted proteins in MSS-CRC (*n* = 7) vs. MSI-CRC (*n* = 6) organoids (adjusted *p* value < 0.1). Significance was based on differential expression analysis using a mixed linear model correcting for replicates and adjusted for multiple testing using the Benjamini-Hochberg method.

Differential expression analysis identified NOTUM, FGF19, COCH, and MFAP2 as the key secreted factors unique to MSS-CRC organoids (Figure 2E; Table S2). NOTUM is a secreted enzyme that normally suppresses Wnt signaling by inactivating Wnt ligands. However, in APC-driven CRC, which is characterized by hyperactive Wnt/β-catenin signaling due to APC mutations, NOTUM becomes upregulated and paradoxically promotes tumor initiation by inhibiting the growth of HC cells.^30^^,^^31^ While COCH has not been associated with CRC biology before, MFAP2 has been shown to promote epithelial to mesenchymal transition.^32^^,^^33^ In addition, MSS-CRC shows increased secretion of FGF19, which can promote liver metastasis by creating a pre-metastatic niche,^34^ in line with the higher incidence of liver metastasis in MSS-CRC. An ELISA for FGF19 and NOTUM, the two top hits discriminating the MSS-CRC vs. MSI-CRC secretome (with NOTUM also a top hit in the MSS-CRC vs. HC secretome) confirmed higher abundance in MSS-CRC organoids (Figure S4D), providing orthogonal protein-level validation for our MS findings.

Together, these data reveal that MSS- and MSI-CRC display fundamentally distinct cancer cell-intrinsic secretory programs, independent of cues from the microenvironment.

### Identification of secreted proteins associated with immune-excluded CRC

Next, we determined whether we could leverage our secretomics dataset to link specific secreted factors to clinically relevant tumor phenotypes. Considering the key role of the secretome in controlling cancer-immune crosstalk, the low immunogenicity of MSS-CRC, and its distinct secretome (Figure 2B), we focused on identifying cancer cell-derived secreted factors that associate with immune-excluded MSS-CRC (Figure 3A).

**Figure 3:**
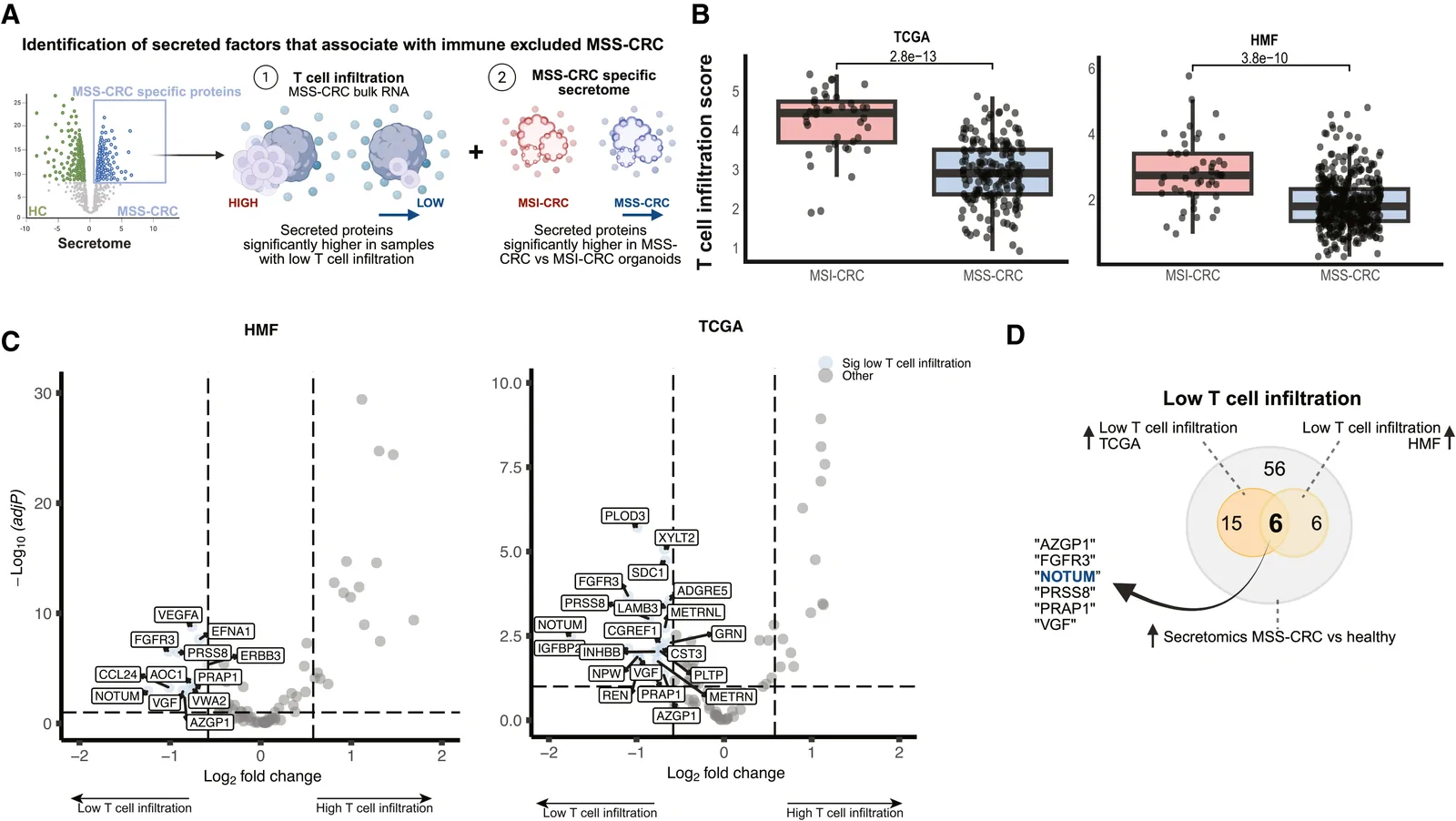
Identification of secreted proteins associated with immune-excluded CRC (A) Workflow to identify secreted proteins associating with immune exclusion in MSS-CRC. First, we performed differential gene expression analysis between MSS-CRC tumors with the top 30% highest T cell infiltration versus the bottom 30% lowest T cell infiltration in bulk RNA sequencing datasets of primary and metastatic CRC from TCGA and HMF. Thereafter, we overlapped genes significantly overexpressed in T cell-poor tumors from both cohorts with our dataset of secreted proteins enriched in MSS-CRC vs. HC and proteins uniquely expressed in the MSS-CRC vs. MSI-CRC secretome. Figure created using Biorender.com. (B) T cell infiltration score in CRC samples in the TCGA (MSI-CRC *n* = 41, MSS-CRC *n* = 242) and HMF (MSI-CRC *n* = 48, MSS-CRC *n* = 432) datasets. *p* value was calculated using Wilcoxon rank-sum test. Boxes, whiskers, and dots indicate quartiles, 1.5 interquartile ranges, and individual tumors, respectively. (C) Volcano plot showing differentially expressed secreted proteins in T cell high vs. T cell low MSS-CRC samples in HMF (*n* = 438) and TCGA (*n* = 242) (adjusted *p* value < 0.1, |log2FC| > 0.58). The HMF and TCGA samples were stratified into the top 30% and bottom 30% quantiles, based on the T cell infiltration score. Analysis was focused on proteins that were significantly upregulated in MSS-CRC vs. HC from Figure 1D. Significance was based on differential expression analysis between these quantiles using limma-voom. (D) Overlap between secreted proteins significantly upregulated in MSS-CRC and significantly associated with low T cell infiltration in CRC samples from the TCGA and HMF cohort. Proteins specifically upregulated in the secretome of MSS-CRC vs. MSI-CRC are marked in bold and blue.

We integrated our secretomics data (Figure 1D) with RNA sequencing datasets of primary and metastatic CRC from TCGA and HMF, respectively (Figure 3A). First, we determined the extent of T cell infiltration using an established T cell signature.^35^^,^^36^ Consistent with previous studies,^36^ both primary and metastatic MSS-CRC show decreased T cell infiltration compared to MSI-CRC (Figure 3B). Nonetheless, the extent of infiltration is highly variable between individual MSS-CRC tumors, with some tumors reaching levels of infiltration matching that of MSI-CRC. We leveraged this heterogeneity to perform differential gene expression analysis (DGE) between MSS-CRC tumors with the top 30% highest T cell infiltration score versus the bottom 30% lowest T cell infiltration score. When comparing genes significantly overexpressed in T cell-poor tumors in both cohorts with our dataset of secreted proteins enriched in MSS-CRC vs. HC organoids (Figure 1D), we identified 6 secreted proteins associated with low T cell activity (Figures 3C and 3D). Given the profound difference in immunogenicity of MSS- and MSI-CRC, we then focused on proteins specific to the MSS-CRC phenotype (Figure 2E). Of the six proteins, NOTUM was also significantly higher in MSS-CRC vs. MSI-CRC organoids, representing a top hit (Figure 3D). NOTUM remained significantly overexpressed in samples with low T cell infiltration after correcting for purity, suggesting the signal is not confounded by higher levels of epithelial cells in immune-excluded tumors (Figure S5A).

These data show the feasibility of identifying secreted factors that associate with clinically relevant phenotypes by cross-referencing tumor-specific and subtype-specific secretomics datasets with clinical cohorts.

### NOTUM is a MSS-CRC specific secreted protein that marks immune-excluded cancers

We identified NOTUM as a prominent secreted factor in multiple analyses: (1) it is one of the most strongly enriched secreted proteins in MSS-CRC vs. HC organoids (Figures 1D and 4A), (2) it is a top hit differentiating the secretory programs of MSS- and MSI-CRC organoids (Figures 2C, 2E, and 4A), and (3) it associates with decreased T cell infiltration in both primary and metastatic MSS-CRC (Figure 4B). Importantly, NOTUM is overexpressed in almost all primary MSS-CRC tumors while very lowly expressed in healthy colorectal tissue (Figure 4C), indicating its tumor specificity. To further investigate the relationship between NOTUM expression and the composition of the TME, we used a third independent single-cell RNA sequencing dataset.^6^ We confirmed that NOTUM is highly upregulated in a subset of epithelial cells in MSS-CRC compared to MSI-CRC and HC. Additionally, NOTUM is specifically upregulated within epithelial cells and not in other cell types within the TME (Figure 4D). When comparing the immune composition of MSS-CRC tumors with the top 35% highest percentage of NOTUM expressing cells (within epithelial cells) to tumors with the bottom 35% lowest percentage of NOTUM expressing cells, we observe significantly lower infiltration of CD4^+^ T cells and NK cells in NOTUM-high tumors, consistent with our results in bulk datasets (Figures 4E and S6A). When testing the effect of NOTUM on T cell reactivity, killing and migration, no direct inhibiting effect was observed (Figures S7A–S7G), suggesting either that NOTUM acts as a marker rather than a direct mediator of immune exclusion, or that it has immunomodulatory roles that are indirect or context dependent and are not captured by these reductionist *in vitro* assays.

**Figure 4:**
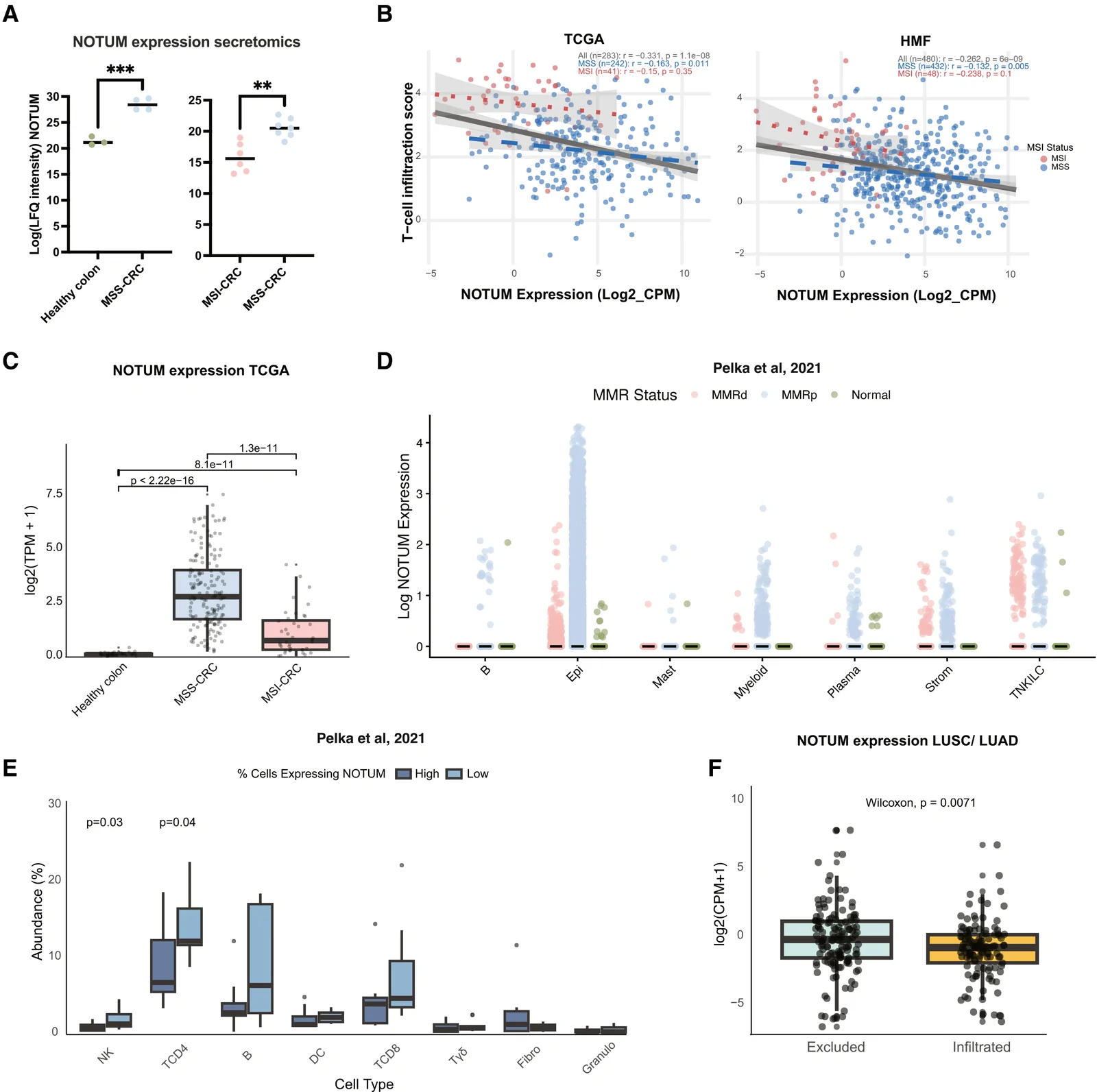
NOTUM is a MSS-CRC specific secreted protein that marks immune-excluded cancers (A) Log_2_(LFQ intensity) of NOTUM in the secretome of MSS-CRC (*n* = 4) vs. HC (*n* = 3) and MSI-CRC (*n* = 6) vs. MSS-CRC (*n* = 7) organoids. *p* values were calculated using unpaired *t* tests (two (∗∗) indicate *p* ≤ 0.01, three (∗∗∗) indicate *p* ≤ 0.001). (B) Pearson correlation between the T cell infiltration score and NOTUM expression in CRC samples from TCGA (MSI-CRC *n* = 41, MSS-CRC *n* = 242) and HMF (MSI-CRC *n* = 48, MSS-CRC *n* = 432). Dots indicate individual tumors. (C) Log_2_ NOTUM expression of NOTUM in MSS-CRC, MSI-CRC and healthy adjacent tissue in TCGA (MSI-CRC *n* = 41, MSS-CRC *n* = 242, HC *n* = 41). Two-sided Wilcoxon rank-sum tests was used to calculate *p* value. Boxes, whiskers, and dots indicate quartiles, 1.5 interquartile ranges, and individual tumors, respectively. (D) Log_2_ NOTUM expression in cell populations in the CRC single cell Pelka et al. 2021 dataset (MSI-CRC *n* = 34, MSS-CRC *n* = 29). Counts were normalized using Normalize Data from Seurat. TNKILC cluster represents T, NK, and ILCs, epi cluster represents epithelial cells and strom cluster represents stromal cells. Dots indicate individual cells. (E) Relative abundance of immune cells within MSS-CRC tumors (*n* = 29) from the CRC single cell Pelka et al. 2021 dataset. Tumors were classified as bottom 35% low or top 35% high NOTUM samples based on percentage of NOTUM expressing cells within epithelial cells. To compare cell type abundances between NOTUM-high and NOTUM-low groups, two-sided Wilcoxon rank-sum tests were performed for each cell type. Boxes, whiskers, and dots indicate quartiles, 1.5 interquartile ranges, and individual tumors, respectively. (F) Log_2_ NOTUM expression in LUAD (*n* = 123) and LUSC (*n* = 135) samples classified as immune infiltrated (LUAD *n* = 54, LUSC *n* = 67) or excluded (LUAD *n* = 69, LUSC *n* = 68). Two-sided Wilcoxon rank-sum test was used to calculate *p* value. Boxes, whiskers, and dots indicate quartiles, 1.5 interquartile ranges, and individual tumors, respectively.

To explore if high expression of NOTUM is also associated with decreased immune infiltration in other cancer types, we performed DGE analysis on a cohort of immune-excluded vs. infiltrated lung adenocarcinoma and squamous cell carcinoma (LUAD and LUSC, respectively) (Table S3). Here, we observe that NOTUM is significantly upregulated in lung tumors with an immune-excluded phenotype (Figures 4F and S6B). This further supports the observation that high expression of NOTUM is associated with decreased immune infiltration.

In conclusion, our data identify NOTUM as a secreted protein strongly upregulated in MSS-CRC that associates with poor T cell infiltration in four independent datasets. These findings provide a foundation for the identification of cancer cell-derived secreted factors that are associated with specific phenotypic states, including immunosuppressive environments. Ultimately, this may pave the way for the development of therapeutic strategies targeting cancer cell-specific secreted factors that interfere with immunosuppressive cell-cell communication and improve responses to cancer immunotherapies.

## Discussion

How cancer cell-intrinsic changes alter the secretome of tumor cells has largely remained unclear due to limitations of conventional secretomics technologies. Here, we integrated patient-derived organoid culture with the metabolic protein labeling method THRONCAT to define the cancer cell-intrinsic secretome of MSS-CRC. While previous studies in CRC have profiled the secretome of bulk tumor samples^10^ or used organoids to profile secretome changes driven by specific cancer drivers,^29^ our approach captures the full genetic complexity of patient tumors by directly comparing patient-derived tumor and HC organoids. This reductionistic system uncouples cancer cell-intrinsic regulation from extrinsic cues and reveals that malignant transformation alone is sufficient to drive extensive rewiring of the secretome. The transition from normal to cancer is associated with cell-autonomous dysregulation of several processes including ECM remodeling, angiogenesis, and neurotrophic factors. Moreover, MSS-CRC organoids display a secretome distinct from the more immunogenic MSI-CRC organoids, indicating that molecular subtypes characterized by divergent tumor microenvironments are also defined by hard-wired differences in the cancer cell-intrinsic secretome. Overall, this positions the cancer cell-intrinsic secretome as a central layer of tumor biology through which cancer cells can shape intercellular crosstalk.

By integrating the cancer cell-intrinsic secretome with multiple clinical cohorts, we were able to link specific secreted factors to clinically relevant phenotypes. Since patients with MSS-CRC are largely resistant to cancer immunotherapies, we focused on cancer cell-derived secreted factors associated with T cell-excluded tumors. Our systematic, secretome-wide analysis revealed NOTUM as the most prominent cancer cell-enriched secreted protein marking tumors with decreased T cell infiltration, recurring across four independent datasets. While previous studies have associated high Wnt signaling and NOTUM expression with immune evasion,^27^^,^^35^^,^^36^^,^^37^^,^^38^ here, we identify NOTUM as the dominant cancer cell-derived secreted factor associated with T cell exclusion in an unbiased manner. NOTUM is one of the most strongly upregulated secreted proteins in the transition from normal to tumor and a top differentiating protein between MSS- and MSI-CRC subtypes. Pharmacological inhibition of NOTUM suppressed tumor growth and metastasis in mouse models, highlighting its therapeutic tractability, although the involvement of the immune system was not assessed in this work.^39^ Recent work using immunocompetent mouse models and murine organoid co-culture systems indicated an immunomodulatory effect of NOTUM.^38^ As our study and reductionist functional assays were not designed to determine mechanistic causality and assess indirect or context-dependent immune effects, further studies are required to determine whether NOTUM, beyond being a marker of immune-excluded tumors, also directly contributes to immune evasion in humans. Yet, the consistent association of NOTUM with immune exclusion across multiple datasets, combined with its overexpression in almost all MSS-CRC tumors while very lowly expressed in healthy tissue, makes it an attractive target for further mechanistic and therapeutic investigation.

We anticipate that our study will be impactful in multiple ways. Our approach lays the foundation for future studies aimed at identifying novel secreted therapeutic targets. First, because organoids can be established with high efficiency directly from patient tissue, they can be directly coupled to clinical data to study changes in secretion during tumor progression (with longitudinal sampling) or therapy response (such as immunotherapy). Second, the high establishment rate of organoids allows capturing the natural diversity of cancers to compare the cancer cell-intrinsic secretome of e.g., primary vs. metastatic tumors at larger scale than previously possible with cell lines.^40^ Third, comparing the cancer-cell intrinsic secretome to the secretome of tumor cells directly *ex vivo* will allow determining which features of the tumor secretome are regulated by tumor-intrinsic vs. -extrinsic factors. In addition, while here we characterize the CRC secretome at baseline, our approach can serve as a modular platform in which the effect of different tumor-intrinsic (e.g., driver mutations) or -extrinsic factors (e.g., hypoxia or IFNy) can be studied individually.^29^ This will allow mapping out the regulators of key secreted factors to identify nodes amenable to therapeutic targeting. Finally, we anticipate that studies that directly integrate secretomics profiling with functional readouts will be most informative. This could for example include comparing the secretome of organoids that differ in their ability to suppress T cell activation or migration, followed by focused CRISPRa overexpression screens in tumor-immune cell co-cultures.^41^ Overall, this will allow understanding of how the MSS-CRC secretome contributes to resistance to cancer immunotherapies.

In summary, we show how the CRC secretome intrinsically differs from that of HC epithelium. We define the secretome independent of microenvironmental influences to understand how the cell-intrinsic changes associated with malignant transformation drive rewiring of secretory programs. This allows distinguishing secreted programs that are initiated and driven by cancer cells, rather than a response to its environment. We anticipate that this provides a framework to map the regulators of secreted factors of interest and provide entry points for therapeutic targeting.

### Limitations of the study

Some limitations of this study warrant discussion. A common challenge in secretome analysis is noise due to release of proteins into the culture media during cellular turnover. We therefore filtered our dataset for proteins annotated as secreted according to the human protein atlas. However, this might also have missed novel biology such as unconventionally secreted proteins, shed membrane proteins, or extracellular vesicle/exosome-associated proteins. Approaches to distinguish active release from cellular debris will be required to identify proteins that are released through non-canonical routes.^42^^,^^43^ Another limitation is the limited number of organoids used in this study, which may have constrained the statistical power of the findings, especially when comparing the two major subtypes of CRC (MSS vs. MSI). With larger cohorts it may become possible to identify heterogeneity at a more granular level and relate secretome profiles to cancer-intrinsic regulators such as genetic profile, sex-associated differences or transcription factor usage. Last, our study has highlighted the association of NOTUM with decreased T cell infiltration. Eventually, any direct immunomodulatory effect of NOTUM will need to be determined experimentally, including *in vivo* studies, since immune cells, stromal components, and vascular networks can influence how secreted proteins act in their native context. Additionally, tumor-derived secreted proteins might act not only locally within the TME but also systemically, which cannot be assessed *in vitro*.

## Resource availability

### Lead contact

Requests for additional information and resources should be addressed to Krijn K. Dijkstra (k.dijkstra@nki.nl).

### Materials availability

No novel reagents were generated as part of this study.

### Data and code availability

• Proteomics data obtained through mass spectrometry have been uploaded to the ProteomeXchange Consortium via the PRIDE repository with the dataset identifier PRIDE: PXD078528. De-identified whole-genome sequencing, clinical metadata, and transcriptomics data provided by the Hartwig Medical Foundation are freely accessible for academic purposes following standard request procedures (Study ID: HMF-DR-366). Access request forms can be found here: https://www.hartwigmedicalfoundation.nl/en/data/data-acces-request/. Single-cell RNA-seq data of colorectal cancer patients provided by Pelka et al.^6^ is freely accessible using GSE178341.
• This paper does not include original code. Bioinformatic analyses of the WGS and transcriptomic data were conducted using open-source tools. References are listed in the key resources table.
• Any additional information required to reanalyze the data reported in this paper are available from the lead contact upon request.

## Acknowledgments

This project was financially supported by the 10.13039/501100021821Oncode Institute to E.E.V. and by an 10.13039/501100003246NWO/ZonMW Veni fellowship to K.K.D. (project number 09150162210100). Unrelated to this study, K.K.D. was supported by a KWF Young Investigator Grant (project number 2025-EXPL/16959). In addition, O.B. was supported by the X-omics Initiative (Project 184.034.019), part of the 10.13039/501100003246NWO National Roadmap for Large-Scale Research Infrastructures. W.S.M.E.T. has received research grants from 10.13039/100004325AstraZeneca and 10.13039/100009857Regeneron.

We thank Ton Schumacher, John Haanen, and Adriaan de Groot for production of MART-1-specific T cells; the NKI genomic core facility and Hartwig Medical Foundation for performing DNA and RNA sequencing; J. Westra and J. Schoep for their help in human tissue collection; L. Hoekman for processing of secretomics samples.

## Author contributions

M.W. contributed to the conceptualization, study design, data collection, formal analyses and writing the manuscript. T.F. contributed to data collection, formal analysis and investigation. J.J.I.C., P.T.v.R., M.C.K., O.B., and J.v.d.H. contributed to data collection. J.J.D. and M.V. contributed to formal analysis, advise on proteomic aspects of the study and review of the manuscript. M.P.-M., A.v.V., K.M., W.S.M.E.T., and D.P. contributed to data collection and formal analysis. B.J.I. and K.B. contributed to advise on proteomic aspects of the study and provision of βES. E.E.V. and K.K.D. contributed to conceptualization, study design, supervision, funding acquisition, review and editing of the manuscript.

## Declaration of interests

E.E.V. is independent nonexecutive director of Sanofi, co-founder of Mosaic Therapeutics, founder and current member of the supervisory board of the Hartwig Medical Foundation and board member and founder of the Center for Personalized Cancer Treatment. W.S.M.E.T. received fees for consulting and advisory roles for Roche and AstraZeneca.

## Declaration of generative AI and AI-assisted technologies in the writing process

During the preparation of this work, the author(s) used ChatGPT for text editing purposes. After using this tool/service, the author(s) reviewed and edited the content as needed and take(s) full responsibility for the content of the published article.

## STAR★Methods

### Key resources table

**Table:** 

REAGENT or RESOURCE	SOURCE	IDENTIFIER
**Antibodies**		

Mouse anti-human MLH1	Roche	M1; Cat#6472966001; RRID:AB_1160581
Mouse anti-human MSH2	Roche	G219-1129; Cat#5269270001; RRID:AB_1160596
Mouse anti-human MSH6	Abcam	EP49; Cat#AC-0047; RRID:AB_11001535
Rabbit anti-human PMS2	Agilent Technologies	EP51; Cat#M3647; RRID:AB_3331634
Mouse anti-human CD3	eBioscience	OKT-3; Cat#16-0037-85; RRID:AB_468855
Mouse anti-human CD28	eBioscience	CD28.2; Cat#16-0289-81; RRID:AB_468926
PE, Hamster Anti-Mouse TCR β Chain	BD Pharmingen	Cat#553172; RRID:AB_394684
BB-700, mouse anti-human CD3	BD Biosciences	Cat#566575; RRID:AB_2860004
BV421, mouse anti-human CD8	BD Biosciences	Cat#562428; RRID:AB_11154035
PE, mouse anti-human CD137	BD Biosciences	Cat#555956; RRID:AB_398477

**Bacterial and virus strains**		

MART-1 TCR retrovirus	Gomez-Eerland et al.^44^	N/A
pLenti CMV Puro Luciferase	Addgene	Cat#17477

**Biological samples**		

Patient derived organoids tumor/healthy (overview in Table 1)	The Netherlands Cancer Institute	NL48824.031.14
Healthy donor blood (buffy coat)	Sanquin blood bank	N/A

**Chemicals, peptides, and recombinant proteins**		

Collagenase II	Sigma-Aldrich	Cat#C6885
Hyaluronidase type IV	Sigma-Aldrich	Cat#H3506
Cultrex RGF Base Membrane Extract (BME) type 2	R&D Systems	Cat#3536-005-02
Primocin	InvivoGen	Cat#ant-pm-2
TrypLE	Gibco	Cat#12604-013
β-ethynylserine (βES)	Provided by Kimberly Bonger, Leiden University, the Netherlands	N/A
Protease inhibitor	Fisher Scientific	Cat#87786
Phosphatase inhibitor	Fisher Scientific	Cat#78420
Azide agarose beads	Sigma Aldrich	Cat#900957
Retronectin	Takara	Cat#T100B
Interleukin-2	Pharmacy Netherlands Cancer Institute	Proleukin
Human IFN-gamma Recombinant Protein	Peprotech	Cat# 300-02-1MG
Trypsin	Gibco	Cat#15090-046
MART-1 WT peptide (EV-10)	ProteoGenix	Cat#1272127
Human recombinant protein NOTUM	MedChem Express	Cat#HY-P79357
Wnt surrogate	Gibco	Cat#PHG0402
NOTUM inhibitor ABC99	MedChem Express	Cat#HY-122832
phorbol-12-myristate-13-acetate	Sigma-aldrich	Cat#19-144
Ionomycin	Sigma-aldrich	Cat#I9657
Prostaglandin E2	Cayman Chemicals	Cat#14010-1
Luciferin	Promega	Cat#E1605
Lymphoprep	Stem Cell technologies	Cat#07811
Lympholyte-H Cell Separation Media	Tebu-bio	Cat#CL5020-R
PHA	Selleck Chemicals GmbH	Cat#S6990
Human CXCL10	Peprotech	Cat#300-12-2506
Collagen I	R&D systems	Cat#3440-005-01
RPMI 1640	GIBCO	Cat#11875093
B27 supplement without vitamin A	GIBCO	Cat#C12587-010
N-Acetylcysteine	Sigma-Aldrich	Cat#A9165-5G
Nicotinamide	Sigma-Aldrich	Cat#N0636
Human recombinant EGF	Peprotech	Cat#AF-100-15
A83-01	Tocris	Cat#2939
SB202190	Cayman Chemicals	Cat#10010399
Prostaglandine E2	Cayman Chemicals	Cat#14010-1
Y-27632	Selleckchem	Cat#S1049
Advanced DMEM/F12	Gibco	Cat#12634-028
Stable Glutamine	Capricorn Scientific	Cat#STA-B
HEPES	GIBCO	Cat#15630-056
Penicillin/streptomycin	GIBCO	Cat#15070063
FCS heat inactivated	Capricorn	Cat#FBS-12A
Human Serum heat inactivated	Sigma-Aldrich	Cat#H3667
Recombinant human IL-7	ImmunoTools	Cat#11340077
Recombinant human IL-15	ImmunoTools	Cat#11340157

**Critical commercial assays**		

MycoAlert™ Mycoplasma Detection Kit	Lonza	Cat#LT07-318
Click-iT Protein Enrichment Kit	Invitrogen	Cat#C10416
CellTiter-Glo 3D Assay	Promega	Cat#G9682
CellTiter-Glo Luminescent Cell Viability assay	Promega	Cat#G7571
DNEasy kit	Qiagen	Cat#69506
RNAeasy kit	Qiagen	Cat#74106
CD8^+^ positive selection Dynabeads	Thermo Fisher Scientific	Cat#11333D
LIVE/DEAD™ Fixable Near IR (780) Viability Kit	Thermo Fisher Scientific	Cat#L34994
FGF19 human ELISA kit	Thermo Fisher Scientific	Cat#EHFGF19
NOTUM human ELISA kit	Abcam	Cat#ab317781

**Deposited data**		

RNA-seq data Hartwig metastatic colorectal cancer patients	Priestley et al.^45^	https://www.hartwigmedicalfoundation.nl/en/data/data-access-request/HMF-DR-366
Clinical metadata Hartwig metastatic colorectal cancer patients	Priestley et al.^45^	https://www.hartwigmedicalfoundation.nl/en/data/data-access-request/HMF-DR-366
COAD RNA-seq data from The Cancer Genome Atlas (TCGA)	NCI Genomic Data Commons (GDC)	https://portal.gdc.cancer.gov/projects/TCGA-COAD
Single-cell RNA-seq analyses of colorectal cancer patients	Pelka et al.^3^	https://www.ncbi.nlm.nih.gov/geo/query/acc.cgi?acc=GSE178341
RNA seq analysis on FFPE material of LUAD and LUSC samples	This paper. (Table S3)	–
Secretomics dataset	This paper. (Tables S1 and S2) Project accession PRIDE: PXD078528	–

**Experimental models: Cell lines**		

HCT-116 (STR profiled)	Laboratory of René Bernards, Amsterdam, the Netherlands	N/A

**Experimental models: Organisms/strains**		

R-spondin producer cell line	Laboratory of Calvin Kuo, Stanford, USA	293T-HA-RspoI-Fc
Noggin producer cell line	Laboratory of Hans Clevers, Utrecht, the Netherlands	HEK293-mNoggin-Fc

**Software and algorithms**		

nf-core/Sarek pipeline (v3.6.1)	Ewels et al.; Garcia et al.^46^^,^^47^	https://github.com/nf-core/sarek
GATK Mutect2 (v4.6.1.0)	Cibulskis et al.^48^	https://gatk.broadinstitute.org/hc/en-us/articles/360037593851-Mutect2
Ensembl Variant Effect Predictor (VEP) (v115.2)	McLaren et al.^49^	https://github.com/Ensembl/ensembl-vep
HMF pipeline oncoanalyser (v2.0)	Watts et al.^50^	https://nf-co.re/oncoanalyser/dev/
Cytoscape (v12.0)	Shannon et al.^51^	https://cytoscape.org
Downstream processing analysis bulk RNA seq data HMF	Battaglia et al.^52^	–
TCGA-Biolinks interface (v2.34.1)	Colaprico A et al.^53^	https://bioconductor.org/packages/release/bioc/html/TCGAbiolinks.html
Gene Set Variation Analysis (GSVA) (v2.4.4)	Hanzelmann et al.^54^	https://master.bioconductor.org/packages/release/bioc/html/GSVA.html
limma-voom (v3.62.2)	Richie et al.^55^	https://bioconductor.org/packages/release/bioc/html/limma.html
Seurat library (v5.3.0)	Hao et al.^56^	https://satijalab.org/seurat/
STAR (v2.7.11b)	Dobin et al.^57^	https://github.com/alexdobin/STAR/releases
DI-ANN (v1.9.2)	Demichev et al.^58^	https://github.com/vdemichev/DiaNN/releases
DI-ANNmv (1.3.2)	Zhang et al.^59^	–
DeSeq2(v1.46.0)	Love et al.^60^	https://bioconductor.org/packages/release/bioc/html/DESeq2.html
FACSDiVa 8.0.2. software	BD Biosciences	https://www.bdbiosciences.com/en-us/products/software/instrument-software/bd-facsdiva-software
FlowJo (v.10.8.2)	FlowJo	https://www.flowjo.com/
R version (v4.2.2)	R core team	www.r-project.org
Prism (v10.0.3)	Graphpad Software	https://www.graphpad.com
Biorender	Biorender	https://www.biorender.com

**Other**		

Amicon Ultra 3kDa cutoff 15mL or 50mL ultrafiltration spin filters	Merck Millipore	Cat#UFC900324; UFC800308
3 μm transwells	VWR	Cat#734-2747

### Experimental model and study participant details

Procedures involving human tissue for organoid derivation were approved by the Medical Ethical Committee of the Netherlands Cancer Institute–Antoni van Leeuwenhoek Hospital (protocol number NL48824.031.14), and written informed consent was obtained from all participants. Baseline characteristics of all participants, including sex and age, are provided in Table 1.

### Method details

#### Organoid establishment and culture

Organoids were generated following the protocol described by Cattaneo et al.^61^ In short, biopsies or surgical resections were obtained from primary and metastatic colorectal cancer (CRC) tissues, as well as from matched healthy adjacent tissues (Table 1). To establish colorectal cancer organoids, the tissue was cut into small fragments (1 mm^3^) and digested using 1.5 mg/ml of collagenase II (Sigma-Aldrich), 10 μg/ml of hyaluronidase type IV (Sigma-Aldrich) and 10 μM Y-27632 (Sigma-Aldrich). To establish healthy colon organoids, the tissue was cut into small fragments and digested using phosphate-buffered saline (PBS) + 12.5 μM EDTA (AccuGENE, cat#51234) or as described previously.^61^ The resulting cell suspensions were resuspended in Cultrex RGF Base Membrane Extract (BME) type 2 (R&D Systems, cat#3536-005-02). To solidify the BME, cells were placed in a 37°C incubator for 15 min before adding complete CRC or HC media (Table S4). During the first two weeks of culture, 1× Primocin (InvivoGen) was supplemented to the medium to prevent microbial contamination. Passaging of organoids was done every 1–2 weeks (depending on growth) by incubating in TrypLE Express (Gibco) for 5–10 min followed by washing in PBS and embedding in BME. Organoid lines were authenticated by single nucleotide polymorphism (SNP) profiling and were routinely screened for Mycoplasma contamination using Mycoplasma PCR43 and the MycoAlert™ Mycoplasma Detection Kit (Lonza, cat#LT07-318). Mismatch repair (MMR) status of the CRC tumors was determined using standard protocols on the original tumors on a Ventana automated immunostainer with the following antibodies: MLH1 clone M1 (Roche), MSH2 clone G219-1129 (Roche), MSH6 clone EP49 (Abcam), and PMS2 clone EP51 (Agilent Technologies).

#### Secretomics

##### Sample preparation

For secretomics, we used the previously published metabolic labeling method THRONCAT.^19^ The different secretomic runs are summarized in Table S5. To collect samples from CRC and HC organoids for secretomics, organoids were first dissociated into single cells and plated at 1.6∗10^6^ cells per replicate in complete CRC or HC culture media, respectively (Table S4). Five days after plating, when organoids had formed again, supernatant was removed and 0.5 mM β-ethynylserine (βES) (provided by Kimberly Bonger, Leiden University, the Netherlands) was added to complete CRC culture media (to avoid confounding effects arising from differences in medium composition) for 24 hrs to metabolically label newly synthesized proteins. After 24 hrs, the supernatant was collected and centrifuged for 5 min at 1,000×*g* to pellet remaining cells. Thereafter, protease inhibitor 1:100 (Fisher Scientific, cat# 87786), and phosphatase inhibitor 1:100 (Fisher Scientific, cat# 78420) were added to the culture media. All samples were stored at -80°C.

Input for secretome analysis was normalized to total protein amount using the Bradford assay. Next, culture media were processed and measured as previously described.^62^ First, conditioned media were concentrated using Amicon Ultra 3kDa cutoff 15mL or 50mL ultrafiltration spin filters (Merck Millipore, cat# UFC900324; UFC800308), according to the manufacturer’s instructions. Resin was prepared by gently resuspending azide agarose beads (Sigma Aldrich, cat#900957) and transferring 100 μL of slurry to 2 mL tubes, followed by adding 900 μL of MilliQ water. The resin was pelleted by centrifugation for 5 min at 1,000*g*, after which the supernatant was carefully aspirated, leaving ∼ 100 μL resin per tube. Media (250 μL) were mixed with 250 μL urea lysis buffer (8M urea, 200 mM Tris pH 8, 4% CHAPS, 1M NaCl), centrifuged at 10,000*g* for 5 min and placed on ice while preparing the 2x catalyst solution. This solution was prepared from components delivered in the Click-iT Protein Enrichment Kit (Invitrogen, cat#C10416), 1 mL 2x solution containing 835 μL milliQ water, 125 μL Reaction Additive 1 (Component D), 20 μL copper (II) sulfate (Component E) and 20 μL Reaction Additive 2 (Component F). The click reaction was set up in the 2 mL-tubes containing 100 μL resin by adding 500 μL medium and 500 μL 2x catalyst solution. βES-labeled proteins were subsequently enriched by rotating the tubes overnight at room temperature with 10 rpm rotation speed. Resin with bound proteins was centrifuged at 1,000*g*, supernants were discarded and resins were washed with 900 μL of MilliQ. Proteins were reduced by resuspending the resin in 0.5 mL SDS wash buffer (1% SDS, 100mM Tris pH 8, 250 mM NaCl and 5mM EDTA) and adding 5 μL of 1M DTT, followed by brief vortexing and heating the tubes for 15 min at 70°C in a heating block. The resin was centrifuged for 5 min at 1,000*g*, supernatants were discarded and resins were resuspended in 0.5 mL iodoacetamide solution (7.4 mg/mL in SDS wash buffer) to alkylate proteins in the dark at room temperature for 30 min. In a tube rack, spin columns (component H of the Click-iT Protein Enrichment Kit) were placed and equipped with valve liners (Supelco 57059). Resin solutions were transferred to the spin columns and resinswashed with (per sample): 20 mL SDS wash buffer, 20 mL 8M urea/100 mM Tris-HCl pH 8, 20 mL 20% isopropanol and 20 mL 20% acetonitrile. After these sets of stringent washes, spin columns were capped with the plugs provided in the kit. Resins were resuspended in 2x 500 μL digestion buffer (100 mM Tris-HCl pH 8, 2 mM CaCl2, 10% acetonitrile) and transferred to 1.5 mL tubes, followed by centrifugation (5 min at 1,000*g*) and aspiration of 800 μL supernatant, leaving ∼ 200 μL digestion buffer in the tubes with the resin. To each sample, 0.5 μg trypsin (Gold, Promega V5280) was added to the slurry, briefly vortexed and incubated at 37°C overnight in a thermoshaker. Resins were pelleted by centrifugation, peptide-containing supernatants were transferred to a clean tube and a subsequent resin wash with 200 μL of LC-MS-grade water was added to the first eluate. Digests were acidified with formic acid (1% final concentration) and desalted by stage tips (Pierce 87784). Eluates were vacuum dried and stored at -80°C until mass spectrometry analysis.

Toxicity of treatment with 0.5 mM βES was evaluated by CellTiter-Glo 3D Assay (Promega, cat#G9682) according to manufacturer’s instructions. In brief, CRC organoids were treated with 0.5mM βES for 24h and 48h, after which cells were lysed and adenosine triphosphate (ATP) was quantified using luminescence to determine the number of viable cells.

##### Mass spectrometry

Peptides were reconstituted in 2% formic acid before analysis on the mass spectrometer. LC-MS/MS was performed by nanoLC-MS/MS on an Orbitrap Astral mass spectrometer (Thermo Scientific) connected to a Vanquish Neo nano-LC system (Thermo Scientific). The Vanquish Neo was operated in the trap-and-elute mode and peptides were loaded onto a Pepmap 100 C18 5μm trap column (300μm x 5mm, Thermo Scientific), before separation on the analytical column (either ES906, 2 μm/150 μm x 150mm, Thermo Scientific, or Aurora Ultimate AUR3-25075C18-TS, 1.7μm/75μm x 25cm, IonOpticks) mounted into an Easyspray ion source (Thermo Scientific) with 1600 V spray voltage applied. The column was heated to 500°C and the flow rate was set to 1.3 μL/min (ES906) or 0.750 μL/min (Aurora) at the start of the method to minimize delay time. Solvent A was composed of 0.1% formic acid/water and solvent B was composed of 0.1% formic acid/80% acetonitrile. Peptides were eluted at a flow rate of 0.8 μL/min (ES906) or 0.4 μL/min (Aurora) in a 41-min (ES906) or 36-min (Aurora) effective gradient, containing a non-linear increase from 8% to 55% or 8 to 45% solvent B, respectively, followed by a 1-min/3 mL/min or 3.5-min/0.750 μL/min wash with 99% solvent B at the end. The column was finally equilibrated using the “fast equilibration” script in combined control mode with a 1450 bar pressure limit. The Orbitrap Astral was run in data-independent acquisition (DIA) mode, with full MS scans being collected in the Orbitrap analyzer with 240,000 resolution at *m*/*z* 200 over a^38^ 0-980 *m*/*z* range. Default charge state was 2+, the normalized AGC target was set to 500% (equivalent to 5e6 charges) and the maximum injection time was 3 ms (ES906) or 5 ms (Aurora). For DIA MS2, a normalized HCD collision energy of 25% was applied to a 380-980 *m*/*z* precursor range using non-overlapping isolation windows of 2Th, with window placement optimization turned on. Scans were acquired in the Astral analyzer over a 100-1000 *m*/*z* range, with the normalized AGC target set to 500% (equivalent to 5e4 charges), a maximum injection time of 2.8 ms (ES906) or 3 ms (Aurora) and a 0.6 sec. cycle time.

##### Data analysis

Raw data were analyzed by DIA-NN (version 1.9.2)^63^ excluding a spectral library and including “Deep learning” option. The Swissprot Human database (DIA-NN 1.9.2: 20,435, release 2024_08) was added for the library-free search. For DIA-NN version 1.8.1 the Robut LC (high accuracy) quantification strategy was used while for DI-ANN version 1.9.2 the QuantUMS (high precision) quanitification strategy was used. All settings were kept at default values except for the MBR option that was enabled. The protein (report.pg_matrix) and peptide (report.pr_matrix) DIANN output were used for downstream analysis using the DIANNmv package in R.^58^ Values were Log2-transformed, after which proteins were filtered for at least 66% valid values in at least one sample group and >1 razor/unique peptide. Missing values were imputed by the ‘man’ method. Next, we filtered out proteins not annotated as secreted according to the human protein atlas, to ensure that the detected proteins are truly secreted rather than released into the culture medium during cellular turnover.^20^ A mixed linear model correcting for replicates was used to identify differentially expressed proteins (minimal threshold: adjusted *p* value ≤0.1).

Pilot data was generated on the Orbitrap Exploris 480 Mass Spectrometer using DDA mode. Downstream analysis was performed in Perseus^59^(version: 2.0.10.0) using the protein groups report from DIA-NN. Values were Log2-transformed, after which proteins were filtered for at least 66% valid values in at least one sample group. Missing values were replaced by imputation based a normal distribution using a width of 0.3 and a minimal downshift of 2.4. Differentially expressed proteins were determined using a Student's t-test (minimal threshold:–log(*p*-value) ≥ 1.3 and [x-y] ≥ 1 | [x-y] ≤ -1).

##### Principal component analysis

Log(LFQ intensities) were used for Principal Component Analysis. To further investigate the contribution of each principal component in separating MSS-CRC vs HC organoids (Figures S3A–S3C) and MSS-CRC vs MSI-CRC organoids (Figures 2B and 2C), we quantified the separation between MSS-CRC vs HC organoids and MSS-CRC vs MSI-CRC organoids along each principal component, using the Cohen's d effect size as the absolute difference in group means divided by the pooled standard deviation. Statistical significance was assessed using two-sided Welch's t-test. To visualize the distribution of principal component scores between MSS-CRC and MSI-CRC organoids, boxplots were generated for the top five principal components. For each PC, samples were grouped by microsatellite instability status.

##### Orthogonal validation secretomics findings

ELISA’s were performed to validate the differential secretion of NOTUM (Abcam, cat #ab317781) in MSS-CRC vs HC and FGF19 (Thermo Fisher Scientific, cat#EHFGF19) in MSS-CRC vs MSI-CRC organoids. To generate conditioned medium, organoids were plated as whole organoids at 2,5∗10^5^ cells/mL for 24h (NOTUM) and 48h (FGF19). Supernatant was collected and centrifuged at 1000*g* for 10 min to remove cellular debris. Conditioned medium was stored at -80°C till further usage during ELISA.

#### DNA sequencing

##### Whole exome DNA sequencing

At least 500 ng of genomic DNA was extracted from CRC organoids and germline blood using a DNEasy kit (Qiagen). After exome capture (Integrated DNA Technologies xGen Exome Research Panel V2.0 or Exome-Agilent_V6), whole-exome sequencing was performed on an Illumina HiSeq DNA or NovaSeq analyzer using 100 bp paired-end reads. WES reads were aligned to the hg38 reference genome using BWA-MEM within the nf-core/Sarek pipeline (v3.6.1).^64^^,^^46^ Somatic single-nucleotide variants (SNVs) and indels were identified using Sarek’s WES workflow. Paired tumor–normal BAM files were provided as input, with the appropriate capture panel specified for each pair, and somatic variant calling was performed with GATK Mutect2 (v4.6.1.0).^47^ All variants were annotated using Ensembl Variant Effect Predictor (VEP) (v115.2).^48^ Results were filtered based on the known annotated driver gene panel reported by the HMF and for mutations predicted to have high or moderate functional impact according to VEP consequence annotations^49^ (Table S6).

##### Whole genome sequencing

For whole genome sequencing, cell pellets of CRC organoids and germline samples (patient matched normal organoids or peripheral blood mononuclear cells (PBMCs) were collected. Samples were sequenced at Hartwig Medical Foundation (HMF), where DNA isolation (QIAGEN) and enzymatic WGS library preparation was performed. Samples were sequenced using the Illumina NovaSeq6000 WGS at 30x coverage for both tumor and germline. Mutation driver calling was done with HMF pipeline oncoanalyser (v2.0).^65^ Somatic variant and copy number drivers are reported in Table S7.

#### STRING analysis

STRING analysis was performed on differential expression output from MSS-CRC vs HC secretomics from the first secretomics run (Table S5). Protein–protein interaction (PPI) networks were generated using Cytoscape based on the STRING db (v12.0),^50^ on protein query mode, with MSS and healthy organoid differential abundance set as input. The differential abundance output nodes were filtered based on a differential log2FC >|0.58|. The resulting network was clustered using the Markov Cluster Algorithm (MCL) with an inflation parameter of 7. The cluster granularity was determined through iterative testing of inflation parameters, selecting the value that yielded stable and biologically interpretable modules without over-fragmentation.

#### Publicly available bulk and single-cell RNA sequencing datasets

Transcriptomic data from metastatic colorectal cancer were obtained from the Hartwig Medical Foundation cohort described by Priestley et al.^51^ Downstream processing analysis was performed as in Battaglia et al.^45^ In addition, primary colorectal adenocarcinoma (COAD) RNA-seq data from The Cancer Genome Atlas (TCGA) were retrieved through the TCGA-Biolinks interface (v2.34.1)^52^ using the Genomic Data Commons (GDC) repository. Single-cell RNA-seq analyses of the MSS colorectal tumor patients were performed using the preprocessed dataset from Pelka et al.^6^ (GSE178341).

#### HMF/TCGA gene set variation analysis

PC2 loadings from the MSI and MSS-CRC secretomics dataset (Table 1; Figures 2B and 2C) were used to derive a negative loading signature since we focused on secreted proteins enriched in MSS-CRC (top 10 genes with highest negative loadings). These signatures were applied to bulk RNA-seq data from TCGA-COAD and HMF colorectal cancer cohorts using Gene Set Variation Analysis (GSVA) (v2.4.4),^53^ which calculates a per-sample enrichment score for each gene set.

#### HMF/TCGA T cell infiltration analysis

##### T cell infiltration score

T cell infiltration scores were calculated for each CRC sample in the HMF and TCGA datasets based on a combined signature of the following genes: CCL2, CCL3, CCL4, CXCL9, CXCL10, CD8A, HLA-DOB, HLA-DMB, HLA-DOA, GZMK, ICOS, and IRF1. This previously established signature has been validated to correlate highly with CD8A expression.^35^^,^^36^ The calculation of the infiltration score is as follows:

$$\text{Tcell}\_\text{infiltration}\_\text{score}=\mu \left(\sum_{i=1}^{n}\log_{2}\left(\text{TP}M_{i}+1\right)\right)$$

TPM_*i*_= Transcript per million normalized expression of infiltration gene *i*; *n*= number of infiltration signature genes; μ(·)= mean.

##### Differential gene expression analysis high versus low T cell infiltration

The HMF and TCGA samples were stratified into the top 30% and bottom 30% quantiles, based on the T cell infiltration score. Differential gene expression between these quantiles was then performed using limma-voom (v3.62.2),^54^ for the identification of genes associated with low T cell infiltration. In this analysis we focused on proteins upregulated in the MSS-CRC secretome vs HC secretome. For the HMF samples, differential gene expression analysis was performed both with and without correction for tumor purity.

##### Correlation analysis NOTUM expression and T cell infiltration

Pearson correlation analyses were performed between voom transformed NOTUM counts and the T-cell infiltration signature score across MSI- and MSS-CRC samples in the TCGA and HMF cohort. Correlations were calculated both within each MSI status group individually and across the combined cohort.

#### Analysis of single cell RNA sequencing data

The single cell RNA sequencing dataset from Pelka et al. was preprocessed using the Seurat library (v5.3.0), retaining cells were filtered for sufficient UMI counts (> 400) and low mitochondrial content (< 25%).^6^^,^^55^ To investigate the relationship between epithelial NOTUM expression and tumor microenvironment composition, we performed cell type abundance analysis stratified by NOTUM expression levels. MSS-CRC samples were stratified based on percentage of NOTUM expressing cells within epithelial tumor cells only. Patients were then classified as NOTUM-high (top 35th percentile) or NOTUM-low (bottom 35th percentile), with the middle 30% excluded from subsequent comparisons. For each patient, we quantified the relative abundance of tumor microenvironment cell populations, including immune cells (γδ T cells, CD8^+^ T cells, CD4^+^ T cells, NK cells, B cells, dendritic cells, and granulocytes) and stromal cells (fibroblasts). Cell type abundances were calculated as the percentage of each cell type relative to the total number of cells per patient. To compare cell type abundances between NOTUM-high and NOTUM-low groups, two-sided Wilcoxon rank-sum tests were performed for each cell type. Statistical significance was defined as *p* < 0.05.

#### NOTUM expression in immune infiltrated and excluded lung tumors

FFPE samples were collected from LUAD and LUSC tumors. Scoring of slides was performed by pathologists, focusing primarily on differences in CD8^+^ T-cell infiltration between the stromal and tumor compartments. Tumors with predominant stromal infiltration and low overall intratumoral CD8^+^ T-cell presence were classified as excluded. RNA was extracted from FFPE samples and sequenced by Q2 solutions, now known as IQVIA labs. Raw read counts were aligned to GrCh38.p13 using STAR (v2.7.11b)^56^ and counted with htseq. Quality control was performed using FastQC. Differential Gene Expression analysis was performed on immune excluded vs infiltrated LUAD and LUSC samples with tumor purity correction using DeSeq2(v1.46.0).^57^ The results were filtered for hits signficantly upregulated in the secretome of MSS-CRC vs HC.

#### T cell killing and reactivity assays

To test the direct effect of NOTUM on T cell reactivity and killing, we performed co-cultures using T cells specific for the MART1 model antigen, a luciferase transduced MSI-CRC cell line and a MSS-CRC organoid line.

##### MART1 specific T cells

MART-1 TCR retrovirus was generated using a packaging cell line following the protocol described by Gomez-Eerland et al.^60^ PBMCs were obtained from healthy donor buffy coats (Sanquin, Amsterdam, The Netherlands) using density gradient centrifugation (lymphoprep, Stem Cell Technologies). CD8^+^ T cells were subsequently isolated using CD8^+^ positive selection Dynabeads (Thermo Fisher Scientific) according to the manufacturer’s instructions. To prime the isolated CD8^+^ T cells, the T cells were resuspended at 1x10^6^ cells/mL in MART1 T cell medium (Table S4) and plated in a 24-well plate coated with plate-bound anti-CD3 and anti-CD28 (5 μg/2x10^6^ cells, eBioscience). After 48 hours, CD8^+^ T cells were resuspended at 1x10^6^ cells/mL in MART1 T cell medium and mixed 1:1 with MART-1 TCR retrovirus. The cell-virus mixture was then spinfected onto a retronectin-coated (Takara, cat#T100B) non-tissue culture treated 24-well plate. After 24 hours, CD8^+^ T cells were collected and maintained in MART1 T cell medium containing 10% fetal bovine serum instead of human serum. MART-1 TCR expression was assessed by flow cytometry one week following transduction (α-mouse TCR β chain, BD Pharmingen, cat#553172).

##### Luciferase transduction

HCT-116 and MSS-CRC organoid luciferase transduced lines were generated by dissociating the tumor cells into single cells and subsequently mixed with pLenti CMV Puro Luciferase at a 1:1 ratio (w168-1) (Addgene_17477). Cells were then spinfected at 600x rpm for 1 hour in a tissue culture treated 48 well plate and incubated overnight at 37°C. After 24h, transduced organoids and HCT-116 cells were selected by puromycin treatment (10 μg/mL) for 2 weeks.

##### Co-cultures

T cells were thawed in prewarmed T cell thawing medium (Table S4) two days before the experiment. After thawing, T cells were cultured at 2∗10^6^ cells/ mL in T cell medium supplemented with 150 U/mL IL-2 (Proleukin) at 37°C. Tumor cells were treated with 200 ng/ml human recombinant IFNγ (Peprotech) one day prior to the experiment. For co-culture, organoids and the cell line were dissociated into single cells using TrypLE Express (GIBCO) and Trypsin (Gibco) respectively. Thereafter, tumor cells were incubated in RPMI medium (without serum) with 1 μg/mL MART-1 WT peptide (EV-10, ProteoGenix cat#1272127) for 30′ at 37°C. Tumor cells were washed twice in PBS and plated together with MART1 T cells in T cell medium supplemented with 150 U/mL IL-2 in a 1:2 E:T ratio in a 96-well U-bottom plate for reactivity assays and white 96-well flat-bottom plate for killing assays. Cells were plated in presence or absence of 500 ng/mL NOTUM (MedChem Express, cat# HY-P79357)), 0.5 nM Wnt surrogate (Gibco, Cat# PHG0402) or 0.05 - 2 μM of the NOTUM inhibitor ABC99 (MedChem Express, cat #HY-122832)). As a positive control for MART1 T cell activation, 50 ng/ml phorbol-12-myristate-13-acetate (Sigma-Aldrich) and 1 μg/ml ionomycin (Sigma-Aldrich) were added. As a control for MART1 T cell suppression, 1 μM prostaglandin E2 (Cayman Chemicals, cat#14010-1) was added to the plate. Cells were co-cultured for 24 hours at 37°C, before measuring T cell reactivity and killing.

To measure T cell reactivity, cells were washed twice in FACS buffer (PBS, 5 mM EDTA, 1% bovine serum antigen (BSA)) and then stained with CD3-BB700 (BD Biosciences, cat#566575), CD8-BV421 (BD Biosciences, cat#562428), CD137-PE (BD Biosciences, cat#555956) and NIR viability dye (Thermo Fisher Scientific, cat# L34994) for 30 min at 4°C. Cells were washed twice and then resuspended in FACS buffer and measured at BD LSRFortessa Cell Analyzer SORP flow cytometer with FACSDiVa 8.0.2. software (BD Biosciences). Data was analyzed using FlowJo (v.10.8.2).

To measure tumor cell viability, 25 μl of 10 mg/mL Luciferin (Promega) was added to the supernatant. Luminescence was measured using a Tecan reader (1000ms exposure).

#### T cell migration assays

T cell migration assay was performed as previously described.^44^ In short, PBMCs were isolated from healthy donor buffy coats (Sanquin, Amsterdam, The Netherlands) using density gradient centrifugation (Lympholyte-H Cell Separation Media, Tebu-bio). To generate effector T cells, PBMCs were resuspended at 2∗10^6^ cells/mL in T cell medium supplemented with 3 μg/ml PHA (Selleck Chemicals GmbH, cat# S6990) and 25 U/mL IL-2 for 3 days, followed by 2 days in the presence of 25 U/mL IL-2 alone. During the last 24 hours of the differentiation protocol, effector T cells were preincubated in the presence or absence of 500 ng/mL NOTUM (MedChem Express, cat# HY-P79357)) and 0.5 nM Wnt surrogate (Gibco, Cat# PHG0402). For the migration assay, CXCL10 (Peprotech, cat# 300-12-2506) was added to the lower wells at 500 ng/ml and effector T cells were added to the upper wells of 3 μm transwells (VWR, cat# 734-2747) coated with 50 μg/ml collagen I (R&D systems, cat# 3440-005-01). Cells were plated in in RPMI 1640 (GIBCO) (no serum, 0.1% BSA, 1% Penicillin/Streptomycin) in the presence or absence of 500 ng/mL NOTUM (MedChem Express, cat# HY-P79357) and 0.5 nM Wnt surrogate (Gibco, Cat# PHG0402). After 4 hrs, T cells that had transmigrated into the lower wells were collected and quantified by using Cell Titer Glo luminescent cell viability kit (Promega, cat# G7571). Luminescence was measured using a Tecan reader (1000ms exposure).

### Quantification and statistical analysis

*p* values were calculated as described in the STAR Methods and figure legends. Only significant comparisons are plotted. One star (∗) is noted for *p* ≤ 0.05, two (∗∗) for *p* ≤ 0.01, three (∗∗∗) for *p* ≤ 0.001, and four (∗∗∗∗) for *p* ≤ 0.0001. “ns” means not significant. Group size, number of replicates and definition of error bars are specified in figure legends. All data was analyzed using R version (v4.2.2) or Prism (v10.0.3).

## Supplemental information

Document S1. Figures S1–S7 and Tables S4 and S5

Table S1. Secretomics MSS-CRC and HC organoids *LFQ intensities filtered*: table showing LFQ intensities for all proteins annotated as secreted according to the Human protein atlas; *Differential expression*: table showing output of differential expression analysis using a mixed linear model. Estimate represents log2foldchange, adj *P* value was calculated using the Benjamin Hochberg method; *LFQ intensities whole dataset*: Table showing LFQ intensities of the unfiltered dataset.

Table S2. Secretomics MSS-CRC and MSI-CRC organoids *LFQ intensities filtered:* table showing LFQ intensities for all proteins annotated as secreted according to the Human protein atlas; *Differential expression:* table showing output of differential expression analysis using a mixed linear model. Estimate represents log2foldchange, adj *p* value was calculated using the Benjamin Hochberg method. *LFQ intensities whole dataset:* table showing LFQ intensities of the unfiltered dataset.

Table S3. RNA sequencing NOTUM LUAD LUSC cohort Raw RNA sequencing counts for NOTUM (raw_counts_NOTUM) from FFPE material from LUAD and LUSC patients (PA_HISTO_TYPE, Patient_ID). Additionally, information about CD8^+^ T cell infiltration status is provided (Category).

Table S6. Whole exome sequencing of CRC organoids Table showing the identified set of somatic single-nucleotide variants and indels in organoids using whole-exome sequencing. For each sample, the dataset includes genomic coordinates (CHROM, POS), reference and alternate alleles (REF, ALT), and gene-level annotations derived from VEP (GENE, SYMBOL). Functional interpretation is provided through VEP-assigned consequence terms (CONSEQUENCE) and predicted impact categories (IMPACT).

Table S7. Whole genome sequencing of CRC organoids Table summarizing the predicted driver alterations derived from somatic variant and copy-number analysis in organoids using whole-genome sequencing samples. For each event, the file reports genomic position (chromosome, chromosome band), affected transcript (transcript), and whether the transcript represents the canonical isoform (isCanonical). Driver classification (driver) is based on the integrated PURPLE hmftools/OncoAnalyzer framework, including variant category (category), likelihood model (likelihoodMethod), and estimated driver likelihood (driverLikelihood). Mutation counts per class (missense, nonsense, splice-site, in-frame, and frameshift variants) are provided alongside biallelic status and the inferred minimum and maximum copy-number states (biallelic, minCopyNumber, maxCopyNumber).
