# Supplementary material for: Cancer cell-intrinsic rewiring of the colorectal cancer secretome
Source: iScience. 2026 Sep 22;29(10):117525. doi: 10.1016/j.isci.2026.117525 (PMC13632250; doi:10.1016/j.isci.2026.117525)
Supplement: Supplementary file 1 [file mmc1.pdf]

iScience, Volume *29*

Supplemental information

Cancer cell-intrinsic rewiring

# of the colorectal cancer secretome

Maartje Witsen, Theodoros Foskolos, Jelmer J. Dijkstra, Miguel Parra-Martinez, Joyce J.I. Catsman, Paula T. van Royen, Bob J. Ignacio, Onno Bleijerveld, Marije C. Kuiken, Alex van Vliet, Kim Monkhorst, Willemijn S.M. E. Theelen, Daniel Peeper, Joris van de Haar, Kimberly Bonger, Michiel Vermeulen, Emile E. Voest, and Krijn K. Dijkstra

SUPPLEMENTAL FIGURE 1

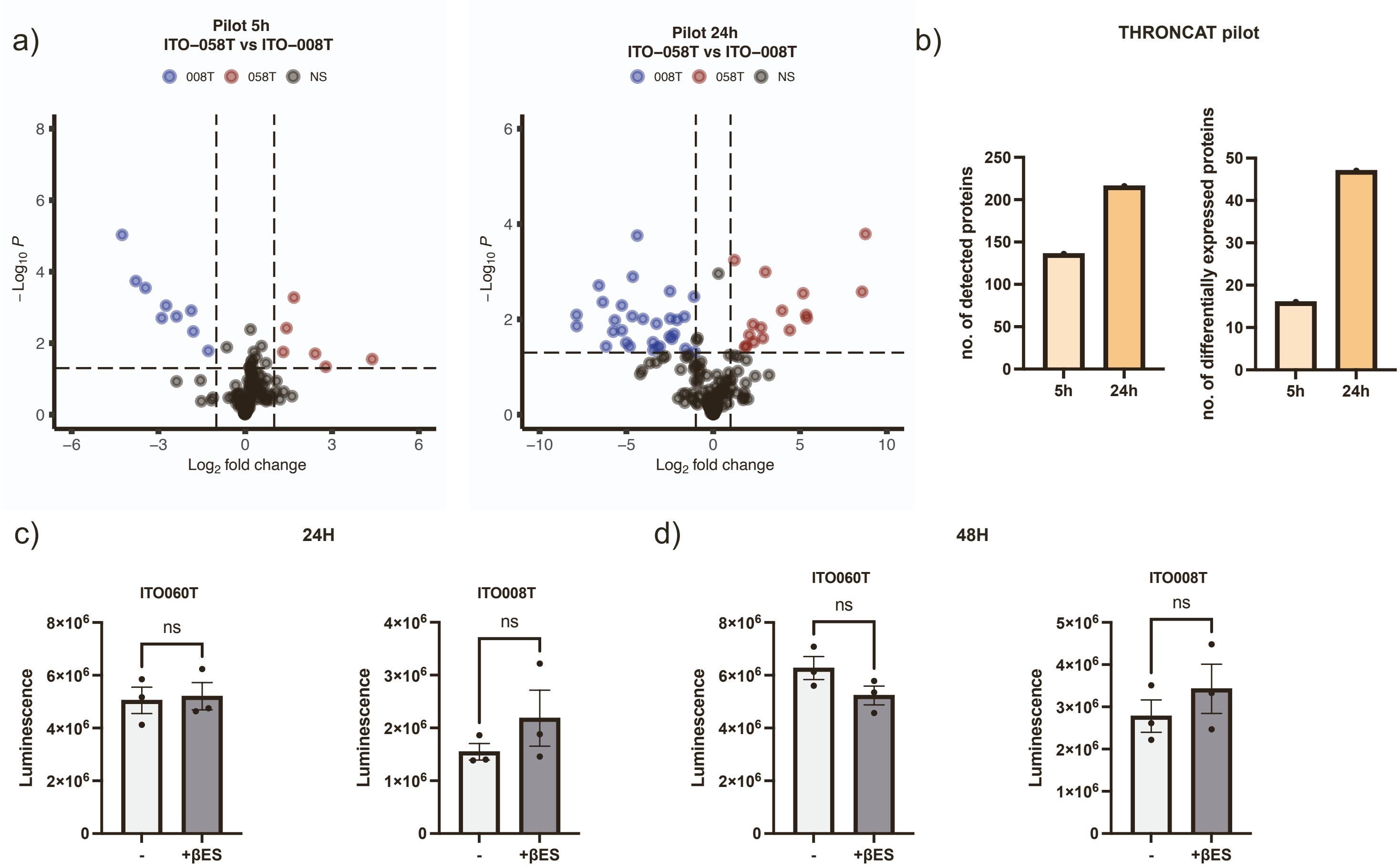

Supplemental Figure 1 Optimization of THRONCAT secretome labeling of CRC organoids, related to Figure 1.

a) Pilot of THRONCAT labeling of CRC secretome at 5h (n=3) and 24h (n=2). Differential expression was performed between MSI-CRC organoid line ITO-058T and MSS-CRC organoid line ITO-008T at 5h and 24h. Differentially expressed proteins were determined using a Student's t-test (minimal threshold:–log(p-value) ≥ 1.3 and log2FC ≥ 1 | log2FC ≤ -1).
b) Total number of detected proteins and differentially expressed proteins of pilot with 5h and 24h THRONCAT labelling. Differentially expressed proteins were determined using a Student's t-test (minimal threshold:–log(p-value) ≥ 1.3 and log2FC ≥ 1 | log2FC ≤ -1).
c) Cell viability of CRC organoids after 24h treatment with 0.5 mM βES quantified by luminescence. Statistical significance was assessed using a paired t-test. Dots indicate mean of technical replicates (n=3). Data are represented as mean (+-SEM).
d) Cell viability of CRC organoids after 48h treatment with 0.5 mM βES quantified by luminescence. Statistical significance was assessed using a paired t-test. Dots indicate mean of technical replicates (n=3). Data are represented as mean (+-SEM).

SUPPLEMENTAL FIGURE 2

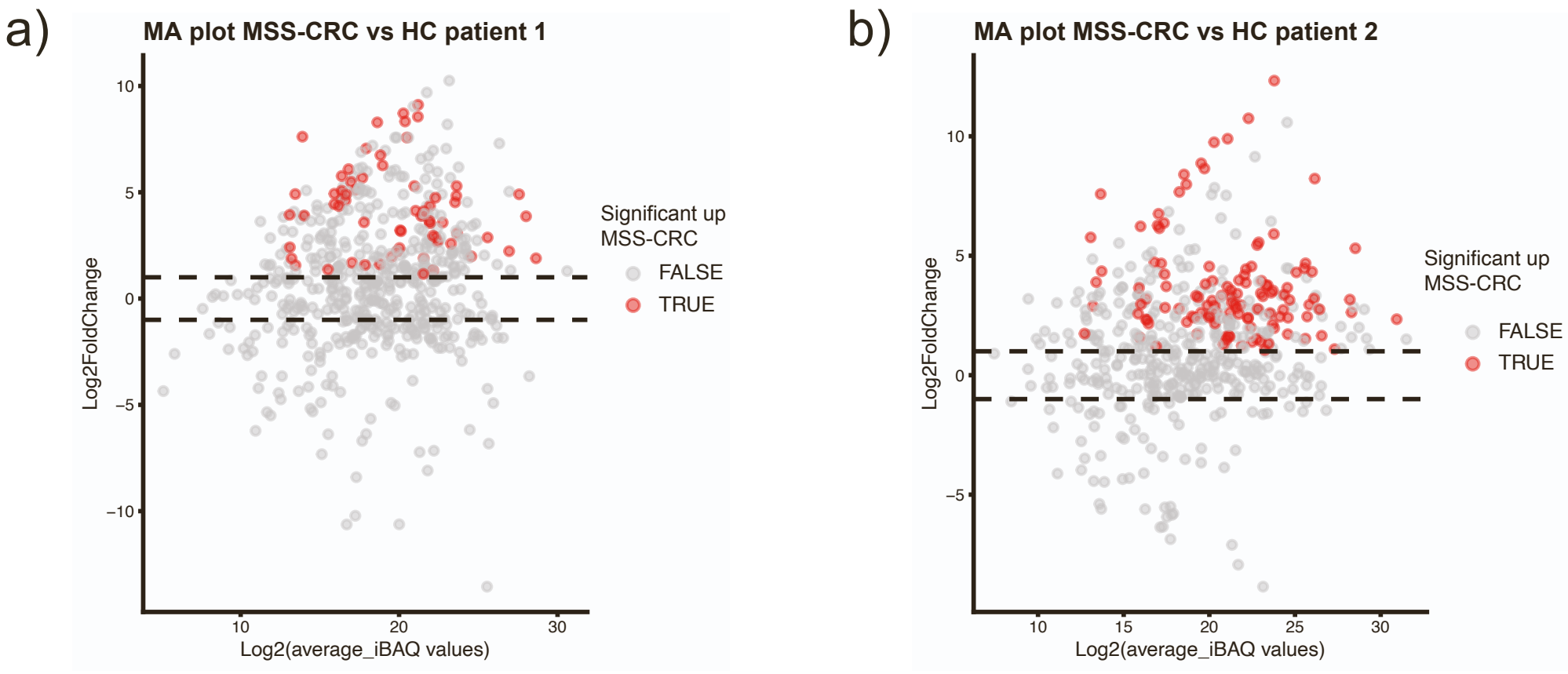

Supplemental Figure 2 MSS-CRC secretome is rewired in matched MSS-CRC and HC organoid pairs, related to Figure 1.

a) MA plot showing log2foldchange and log(average iBAQ values) of secreted proteins in MSS-CRC vs HC organoids from patient 1 (two independent replicates per organoid line). Proteins that are significantly upregulated in MSS-CRC are marked in red ($log2FC > 1$, $adjP < 0.05$). Significance was based on differential expression analysis using Student's t-test and adjusted for multiple testing using the Benjamini-Hochberg method.
b) MA plot showing log2foldchange and log(average iBAQ values) of secreted proteins in MSS-CRC vs HC organoids from patient 2 (two independent replicates per organoid line). Proteins that are significantly upregulated in MSS-CRC are marked in red ($log2FC > 1$, $adjP < 0.05$). Significance was based on differential expression analysis using Student's t-test and adjusted for multiple testing using the Benjamini-Hochberg method.

SUPPLEMENTAL FIGURE 3

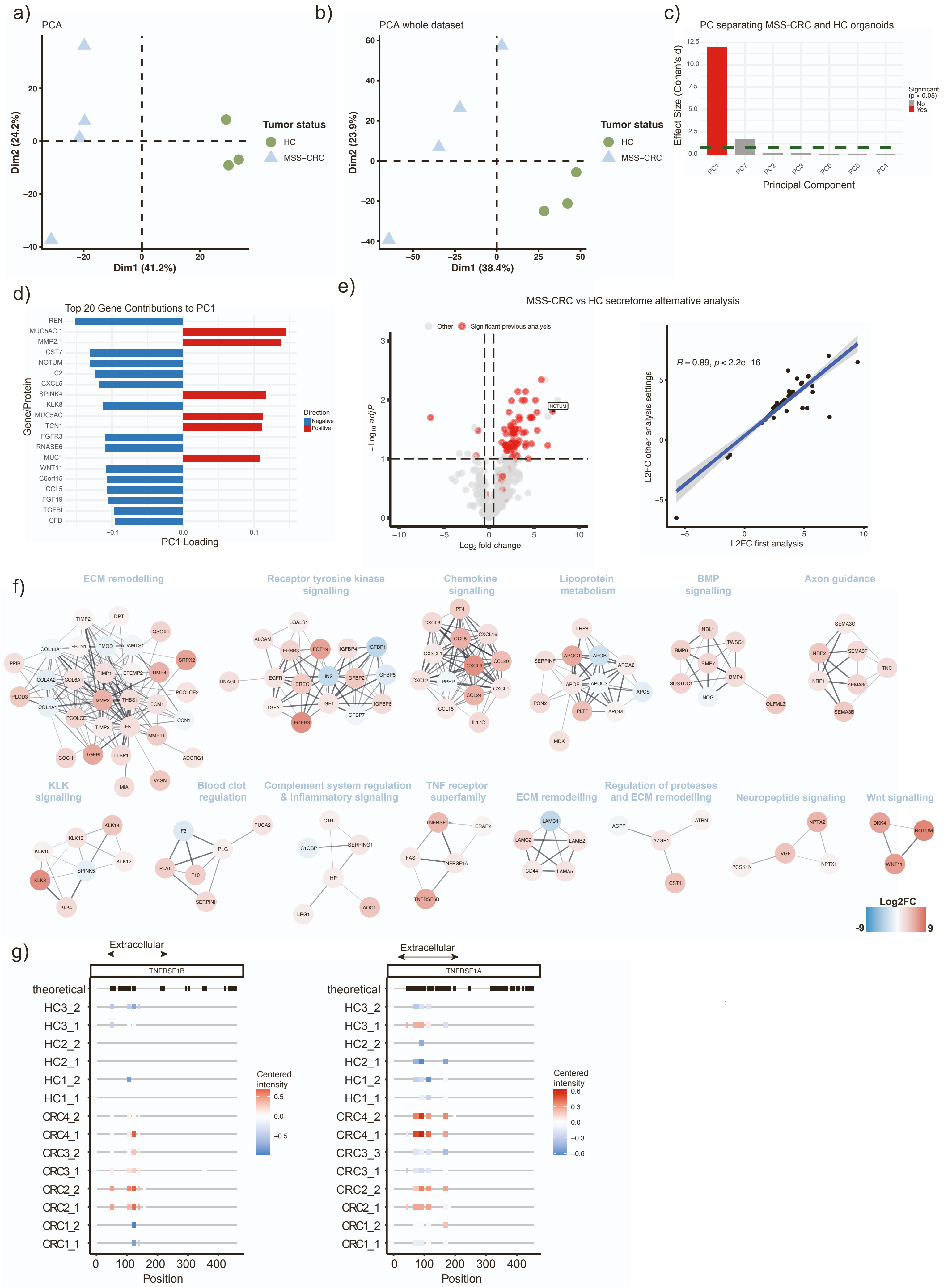

Supplemental Figure 3 In depth-analysis of the MSS-CRC vs HC secretome, related to Figure 1.

a) PCA showing the first two principal components based on the secretomics values from HC (n=3) and MSS-CRC organoids (n=4) (average from two independent replicates per organoid line).
b) PCA showing the first two principal components based on the secretomics values not filtered for annotated secreted proteins from HC (n=3) and MSS-CRC organoids (n=4) (average from two independent replicates per organoid line).
c) Effect size (Cohen's d) for best separation of MSS-CRC and HC organoids for each principal component in Supplemental Figure 3A. Statistical significance was assessed using two-sided Welch's t-test.
d) Top 20 genes contributing to variance to PC1 in the PCA in Supplemental Figure 3A. Negative contributors are marked in blue and positive contributors are marked in red.
e) Differential expression and Pearson correlation analysis of MSS-CRC vs HC organoids with alternative analysis settings; including a stricter protein inclusion criterion requiring a minimum of three razor/unique peptides per protein, as well as a different imputation strategy for handling missing values ('a mixed strategy', which uses both missing-not-at-random (MNAR) and missing-at-random imputation (MAR)). Significance was based on differential expression analysis using a mixed linear model correcting for replicates and adjusted for multiple testing using the Benjamini-Hochberg method. Proteins significant in the original analysis are higlighted in red. For the correlation analysis comparing logFC between original and alternative analysis settings, only proteins significant in the original analysis are shown.
f) STRING protein interaction network on the secretomics dataset MSS-CRC (n=4) vs HC (n=3) organoids. Color indicates log2 fold change. Only nodes with log2 fold change > 0.58 and clusters with at least 4 members are shown.
g) Coverage and intensity of identified peptides for TNFRSF1B and TNFRSF1A in MSS-CRC and HC organoids. Extracellular domain of the proteins is indicated with an arrow.

SUPPLEMENTAL FIGURE 4

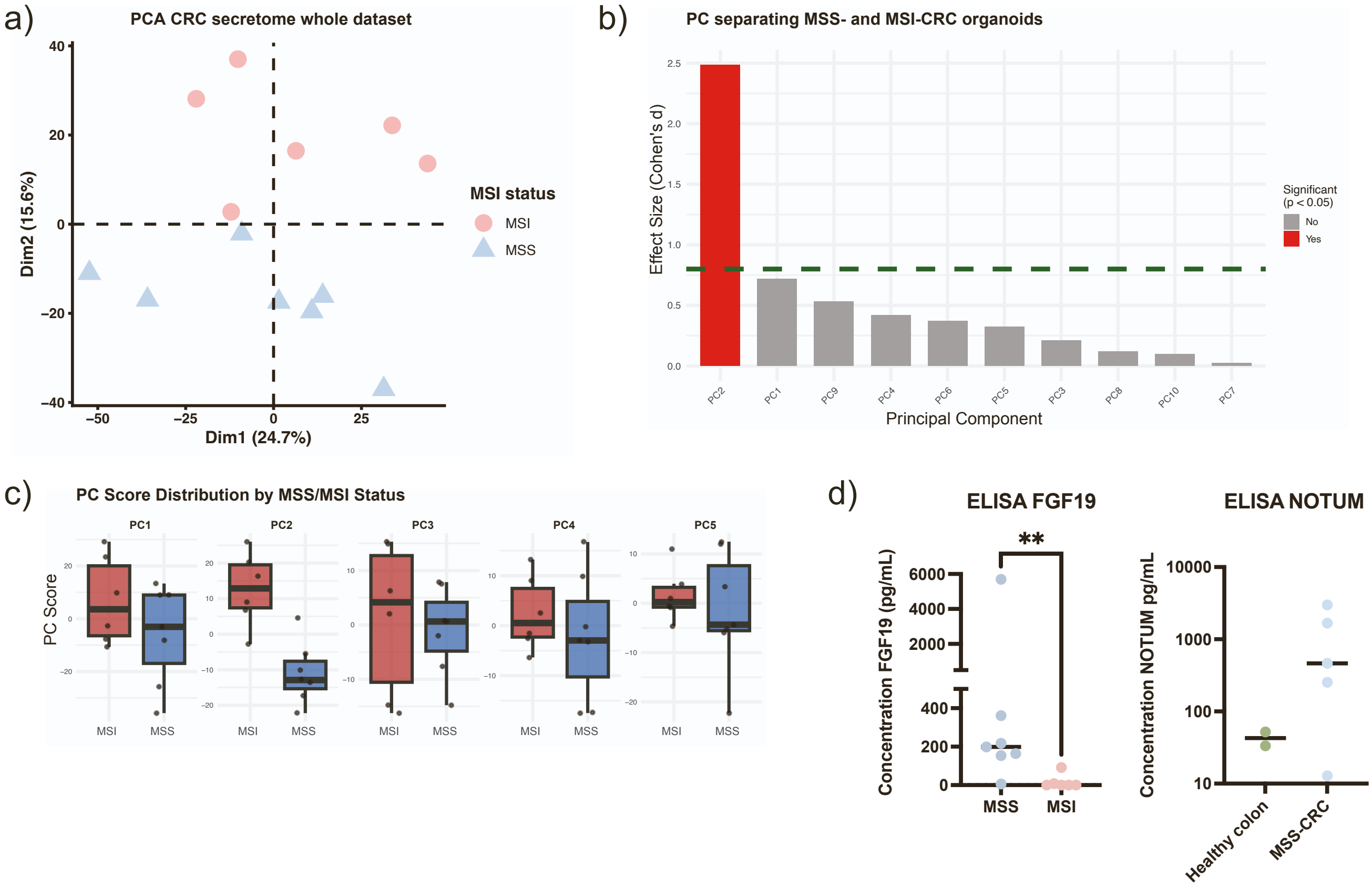

Supplemental Figure 4 PC analysis MSS- and MSI-CRC secretome and orthogonal protein validation, related to Figure 2.

a) PCA showing the first two principal components based on the secretomics values not filtered for annotated secreted proteins from MSS-CRC (n=7) and MSI-CRC (n=6) organoids (average from three independent replicates per organoid line).
b) Effect size (Cohen's d) for best separation of MSS- and MSI-CRC organoids for each principal component in Figure 2B. Statistical significance was assessed using two-sided Welch's t-test.
c) PC score distribution in MSS and MSI-CRC organoids from PC analysis in Figure 2B. Boxes, whiskers, and dots indicate quartiles, 1.5 interquartile ranges, and individual CRC organoids, respectively.
d) FGF19 and NOTUM ELISA analysis performed on conditioned medium of MSS-CRC (n=7) vs MSI-CRC organoids (n=6) and MSS-CRC (n=5) vs HC organoids (n=2), respectively. Each dot represents the average of two independent replicates of conditioned medium for each organoid line. For FGF19, statistical significance was assessed using Mann-Whitney U test, two (**) indicate $p \leq 0.01$

SUPPLEMENTAL FIGURE 5

a)

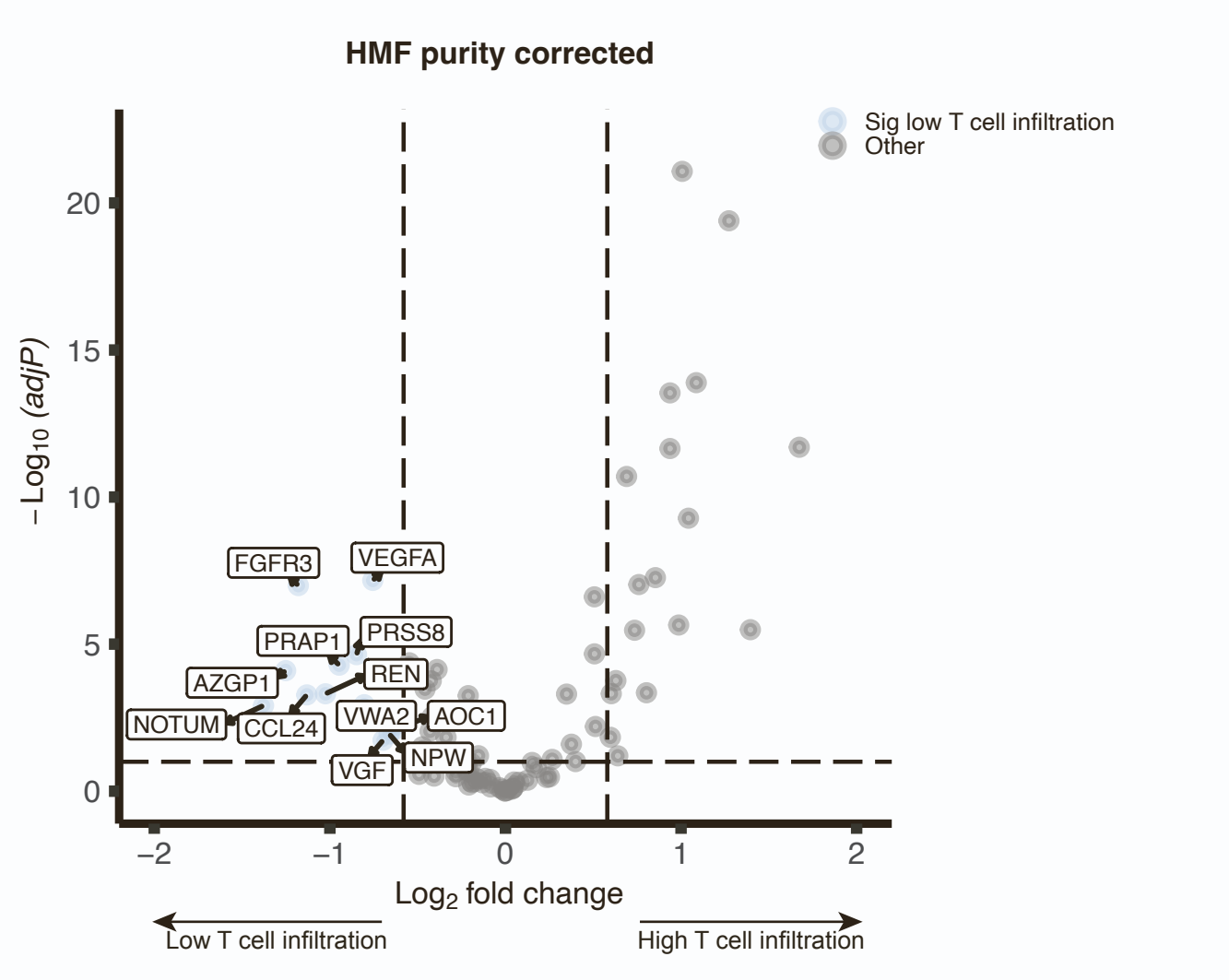

Supplemental Figure 5 Purity corrected differential gene expression analysis high vs low T cell infiltrated MSS-CRC tumors, related to Figure 3.

a) Volcanoplot showing differentially expressed secreted proteins in T cell high vs T cell low MSS-CRC samples (n=438) in HMF (adjusted p value <0.1, log2 fold change > 0.58) while corrected for tumor purity. HMF samples were stratified into the top 30% and bottom 30% quantiles, based on the T cell infiltration score. Analysis was focused on proteins that were signficantly upregulated in MSS-CRC vs HC from figure 1D. Significance was based on differential expression analysis between these quantiles using using limma-voom.

SUPPLEMENTAL FIGURE 6

a)

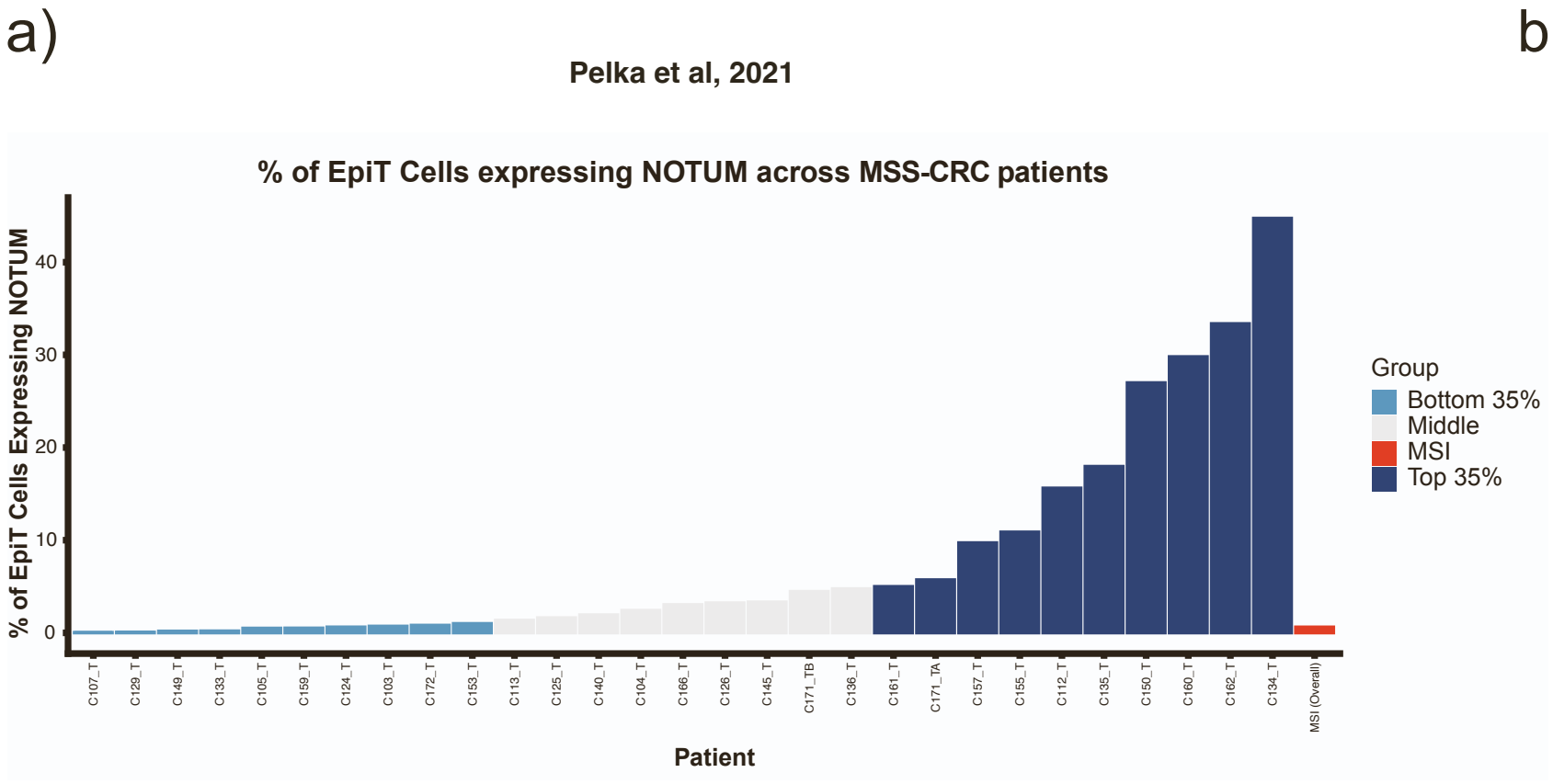

b)

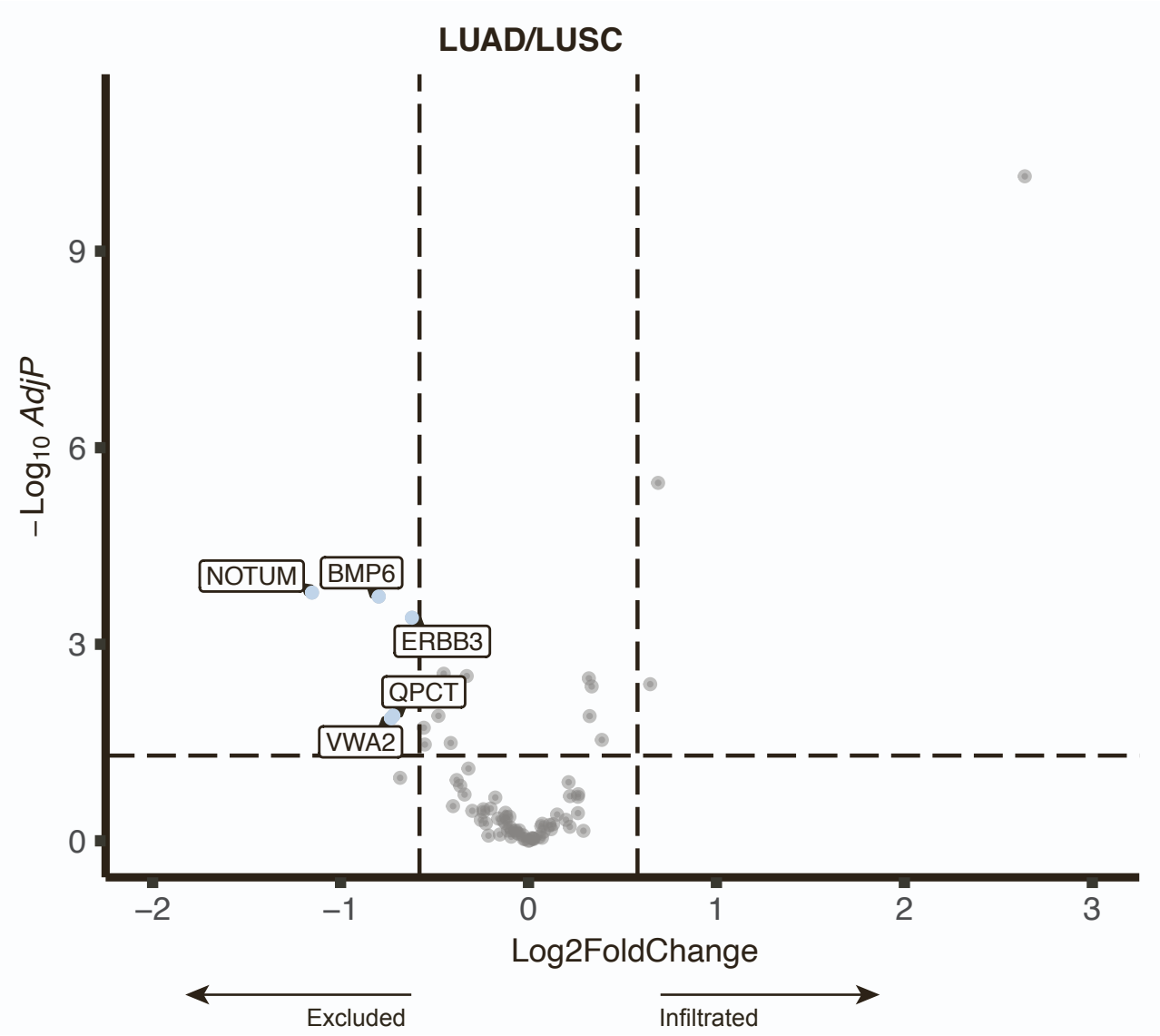

Supplemental Figure 6 NOTUM expression in a single cell RNA sequencing dataset of MSS-CRC and bulk RNA sequencing dataset of immune excluded LUAD/LUSC, related to Figure 4.

a) Percentage of NOTUM expressing epithelial cells across MSS-CRC samples (n=29) in Pelka et al 2021. TA and TB indicate different biopsies from the same patient. Top 35% samples with highest NOTUM expression are marked in dark blue and bottom 35% samples are marked in light blue. Mean percentage of NOTUM expressing epithelial cells across MSI-CRC samples samples is shown in red.
b) Volcano plot showing differentially expressed secreted proteins in infiltrated vs excluded LUSC and LUAD samples (adjusted p value <0.1, log2 fold change > 0.58) while corrected for purity. Analysis was focused on proteins that were signficantly upregulated in MSS-CRC vs HC from figure 1D. Significance was based on differential gene expression analysis on immune excluded vs infiltrated LUAD and LUSC samples using DeSeq2.

SUPPLEMENTAL FIGURE 7

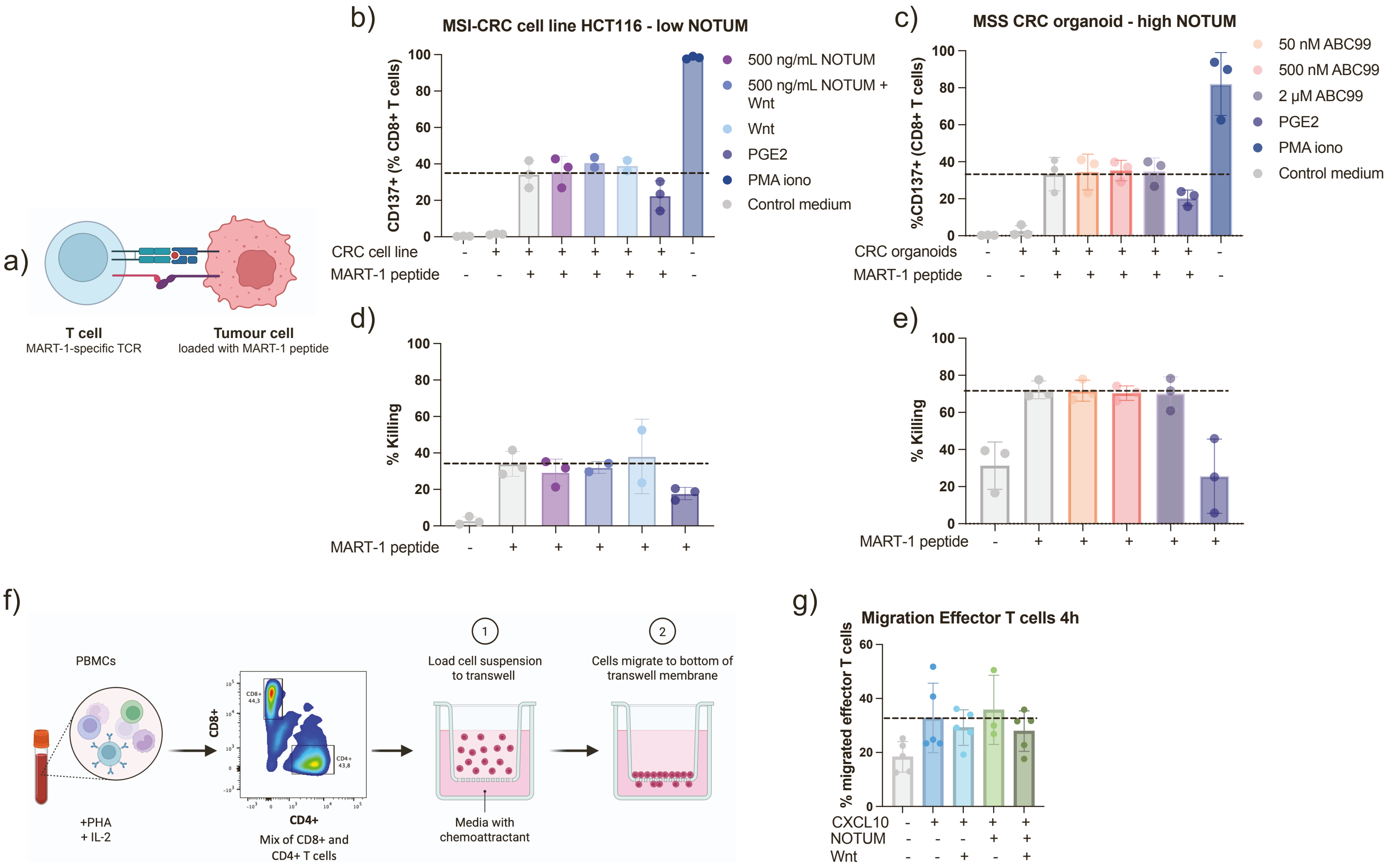

Supplemental Figure 7 No direct immunomodulatory effect of NOTUM in *in vitro* culture assays, related to Figure 4.

a) Overview of MART-1 matched antigen TCR system. Figure created using biorender.com.
b) Reactivity of MART-1 specific T cells towards HCT-116 cells loaded with MART-1 peptide in presence or absence of NOTUM and/or 0.5 nM Wnt. Data are presented as mean with SD. Dots indicate mean of technical replicates (n=3).
c) Reactivity of MART-1 specific T cells towards a MSS-CRC organoid line loaded with MART-1 peptide in presence or absence of NOTUM inhibitor ABC99. Data are presented as mean with SD. Dots indicate mean of technical replicates (n=3).
d) Percentage (%) killing of HCT-116 cells loaded with MART-1 peptide by MART-1 specific T cells in presence or absence of NOTUM and/or 0.5 nM Wnt. PGE2 served as a positive control. Data are presented as mean with SD. Dots indicated mean of technical replicates (n=3).
e) Percentage (%) killing of MSS-CRC organoid line loaded with MART-1 peptide by MART-1 specific T cells in presence or absence of NOTUM inhibitor ABC99. Data are presented as mean with SD. Dots indicate mean of technical replicates (n=3).
f) Overview of effector T cell migration assay. Figure created using biorender.com.
g) Percentage of migrated effector T cells after 4 hours in presence or absence of CXCL10, 500 ng/mL NOTUM and/or 0.5 nM Wnt. Data are presented as mean with SD. Each dot indicates a different PBMC donor (n=5).

Supplementary Tables

Supplementary Table 4: composition of culture media, related to Star Methods.
Overview of culture media used in this study.

Ad-DF+++ media

Reagent	Supplier	Cat no.	Final concentration
Advanced DMEM/F12	Gibco	12634-028	
Stable Glutamine	Capricorn Scientific	Cat#STA-B	2 mM
HEPES	GIBCO	Cat#15630-056	10 mM
Penicillin/streptomycin	GIBCO	15070063	100 U/mL

Complete **normal colon** organoid medium with **Wnt surrogate – Fc Fusion protein***

Reagent	Supplier	Cat no.	Final concentration
Noggin-conditioned medium	In house		10%
R-spondin1-conditioned medium	In house		20%
Wnt surrogate – Fc Fusion protein	Gibco	PHG0402	0.5 nM
Ad-DF+++	See above		70%
B27 supplement without vitamin A	Invitrogen	12587-010	2%
N-Acetylcysteine	Sigma-Aldrich	A9165-5G	1.25 mM
Human recombinant EGF	BD Bioscience	354052	50 ng/mL
Nicotinamide	Sigma-Aldrich	N06636	10 mM
A-83-01	Tocris	2939	0.5 µM
SB202190	Cayman Chemicals	10010399	3 µM
Prostaglandine E2	Cayman Chemicals	14010-1	10 nM
Y-27632<br>*NB: only when passaging, starting up culture, or after thawing*	Selleckchem	S1049	10 µM
Primocin<br>*NB: Add to the medium for the first 2 weeks of culturing after isolation form biopsy/surgical specimen.*	Invivogen	ant-pm-2	

** Prepared medium can only be used up to 1 week if kept at 4C.*

Complete **colorectal cancer** organoid medium*

Reagent	Supplier	Cat no.	Final concentration
Noggin-conditioned medium	In house		10%
R-spondin1-conditioned medium	In house		20%
Ad-DF+++	See above		70%
B27 supplement without vitamin A	Invitrogen	12587-010	2%
N-Acetylcysteine (NAC)	Sigma-Aldrich	A9165-5G	1.25 mM
Human recombinant EGF	Peprotech	AF-100-15	50 ng/mL
Nicotinamide	Sigma-Aldrich	N0636	10 mM
A-83-01	Tocris	2939	0.5 µM
SB202190	Cayman Chemicals	10010399	3 µM
Prostaglandine E2	Cayman Chemicals	14010-1	10 nM
Y-27632<br>*NB: only when passaging, starting up culture, or after thawing.*	Selleckchem	S1049	10 µM
Primocin<br>*NB: Add to the medium for the first 2 weeks of culturing after isolation form biopsy/surgical specimen.*	Invivogen	ant-pm-2	

** Prepared medium can only be used up to 2 weeks if kept at 4C.*

T cell thawing medium

Reagent	Supplier	Cat no.	Final concentration
RPMI 1640	GIBCO	11875093	
Stable Glutamine	Capricorn Scientific	Cat#STA-B	2 mM
FCS heat inactivated	Capricorn	FBS-12A	10%
Penicillin/Streptomycin	Invitrogen	15140-122	100 U/mL

T cell medium

Reagent	Supplier	Cat no.	Final concentration
RPMI 1640	GIBCO	11875093	
Stable Glutamine	Capricorn Scientific	Cat#STA-B	2 mM
Human Serum heat inactivated	Sigma-Aldrich	H3667	10%
Penicillin/Streptomycin	Invitrogen	15140-122	100 U/mL

MART1 T cell medium

Reagent	Supplier	Cat no.	Final concentration
RPMI 1640	GIBCO	11875093	
Stable Glutamine	Capricorn Scientific	Cat#STA-B	2 mM
Human Serum heat inactivated	Sigma-Aldrich	H3667	10%
Penicillin/Streptomycin	Invitrogen	15140-122	100 U/mL
IL-2	Proleukin, Novartis		100 U/mL
IL-7	ImmunoTools	11340077	10 ng/mL
IL-15	ImmunoTools	11340157	10 ng/mL

Supplementary Table 5: Overview of different secretomics runs, related to Figure 1&2 and Star Methods. Overview of which organoids are included in the different secretomics runs.

**Secretomics run**	**HC samples**	**MSS-CRC samples**	**MSI-CRC samples**	**Description**	**Analyses**
**1**	ITO-063H<br>ITO-113H<br>ITO-117H	ITO-008T<br>ITO-60T<br>ITO-063T<br>ITO-113T	-	MSS-CRC vs HC	Figure 1
**2**	-	ITO-008T<br>ITO-014Tb<br>ITO-60T<br>ITO-063T<br>ITO-108T<br>ITO-113T<br>ITO-179T	ITO-045T<br>ITO-058T<br>ITO-058M<br>ITO-079T<br>ITO-111Ton<br>ITO-111Tpre	MSS-CRC vs MSI-CRC	Figure 2
